# Supplementary material for: Genomic insights into local adaptation and future climate-induced vulnerability of a keystone forest tree in East Asia
Source: Nat Commun. 2022 Nov 1;13:6541. doi: 10.1038/s41467-022-34206-8 (PMC9626627; doi:10.1038/s41467-022-34206-8)
Supplement: Supplementary file 1 — Supplementary Information [file 41467_2022_34206_MOESM1_ESM.pdf]

Supplementary Information

Genomic insights into local adaptation and future climate-induced vulnerability of a keystone forest tree in East Asia

Sang et al.

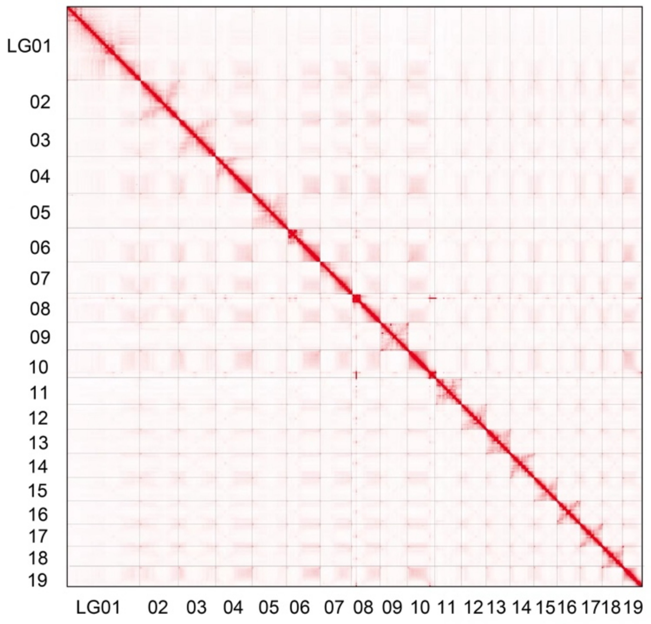

Supplementary Fig. 1. Hi-C heatmap showing chromatin interactions at 100 kbp resolution in the genome assembly of *P. koreana*.

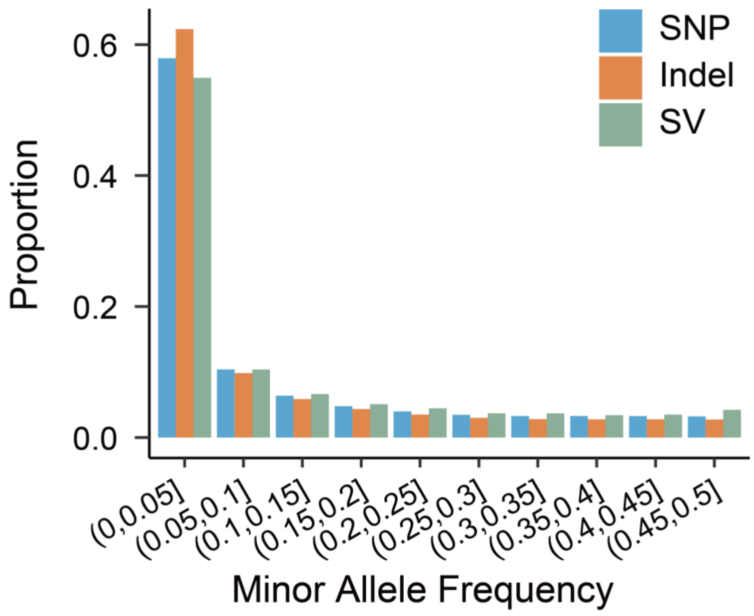

Supplementary Fig. 2. The distribution of minor allele frequency of SNPs, indels and SVs for all *P. koreana* individuals.

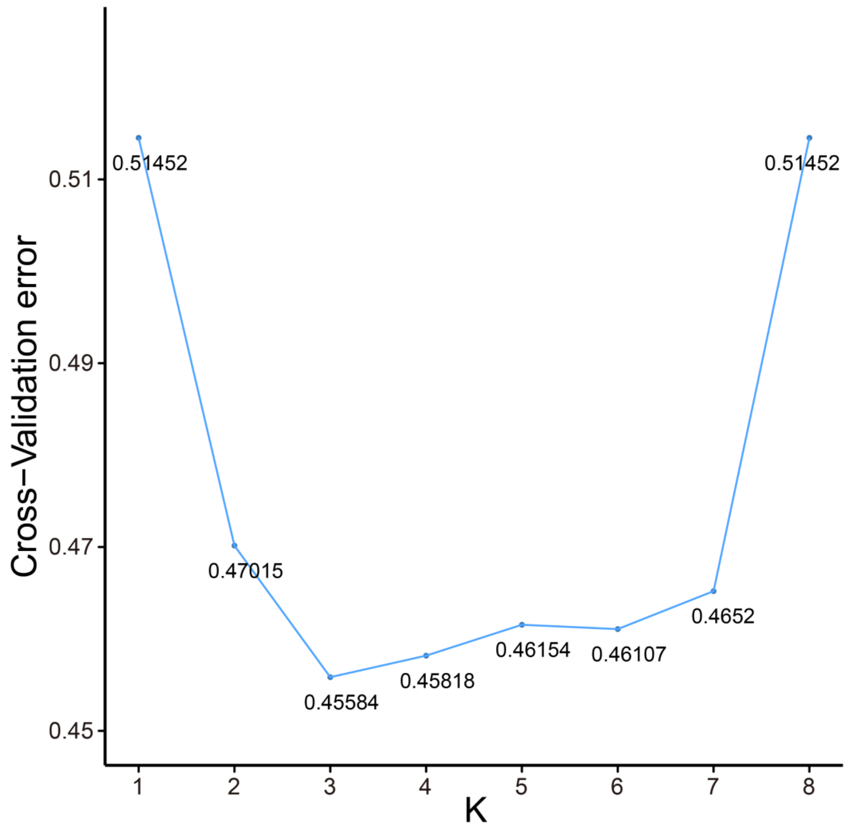

Supplementary Fig. 3. The Cross-Validation error distribution according to the number of clusters (K) by ADMIXTURE.

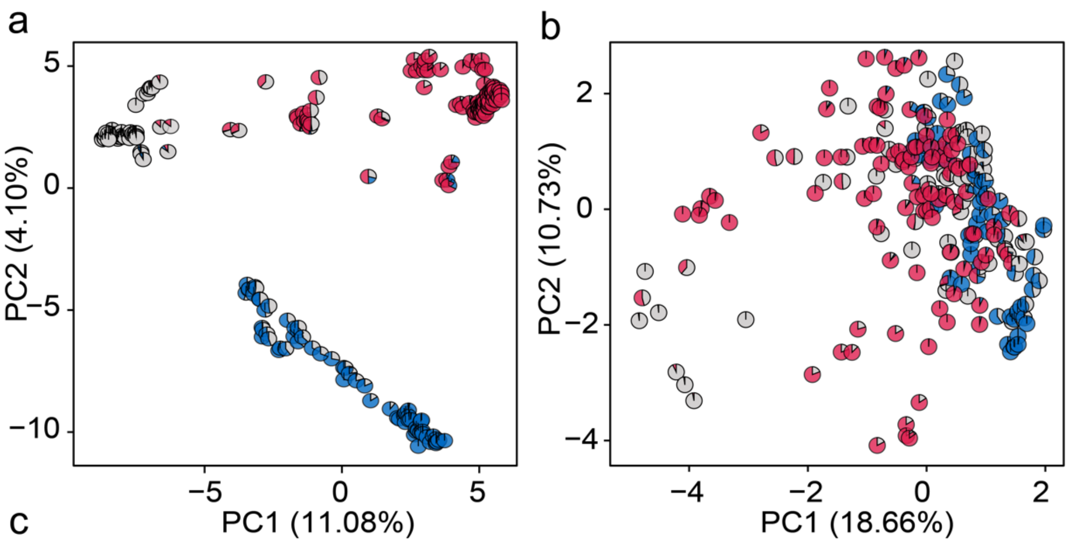

c

Partial RDA models	Inertia	R^2(adj)	*p* (>F)	PEV	PTV
Full model: *F* ~ *clim.* + *geog.* + *struct.*	4546.54	0.528	0.001***	1.00	0.53
Full model: *F* ~ *clim.*|(*geog.* + *struct.)*	896.87	0.104	0.182	0.20	0.10
Full model: *F* ~ *struct.*|(*clim.* + *geog.*)	581.42	0.067	0.258	0.13	0.07
Full model: *F* ~ *geog.* |(*clim.*+ *struct.)*	386.71	0.045	0.487	0.09	0.04
Confounded climate/structure/geography	2681.54			0.58	0.32
Total unexplained	4070.96				0.47
Total inertia	8,617.50				1.00

d

Partial RDA models	Inertia	R^2(adj)	*p* (>F)	PEV	PTV
Full model: *F* ~ *clim.* + *geog.* + *struct.*	66.53	0.827	0.001***	1.00	0.83
Full model: *F* ~ *clim.*|(*geog.* + *struct.)*	33.12	0.412	0.021*	0.50	0.41
Full model: *F* ~ *struct.*|(*clim.* + *geog.*)	2.25	0.028	0.391	0.03	0.03
Full model: *F* ~ *geog.* |(*clim.*+ *struct.)*	4.26	0.053	0.402	0.06	0.05
Confounded climate/structure/geography	26.90			0.41	0.34
Total unexplained	13.88				0.17
Total inertia	80.41				1.00

PEV: The proportion of explainable variance
PTV: The proportion of total variance

****p* ≤ 0.001; ***p* ≤ 0.01; **p* ≤ 0.05

Supplementary Fig. 4. Comparison of PCA and partial-RDA analyses for the neutral and adaptive variants. **a,b,** The principal component analysis estimated based on the neutral variants (**a**, the 535,191 linkage disequilibrium-pruned independent SNPs used for population structure analysis) and the potentially adaptive variants (**b**, the 1,779 adaptive variants identified by both LFMM and RDA). The composition of each pie based on the ancestral components inferred by ADMIXTURE (according to the substructure at *K* = 3; **Fig. 2a**). **c,d,** The relative contribution of climate, geography and neutral genetic structure in shaping neutral (**c**) and adaptive (**d**) genetic variation as estimated with pRDA (partial redundancy analysis). Inertia is analogous to variance and *P* values are calculated from *F*-test.

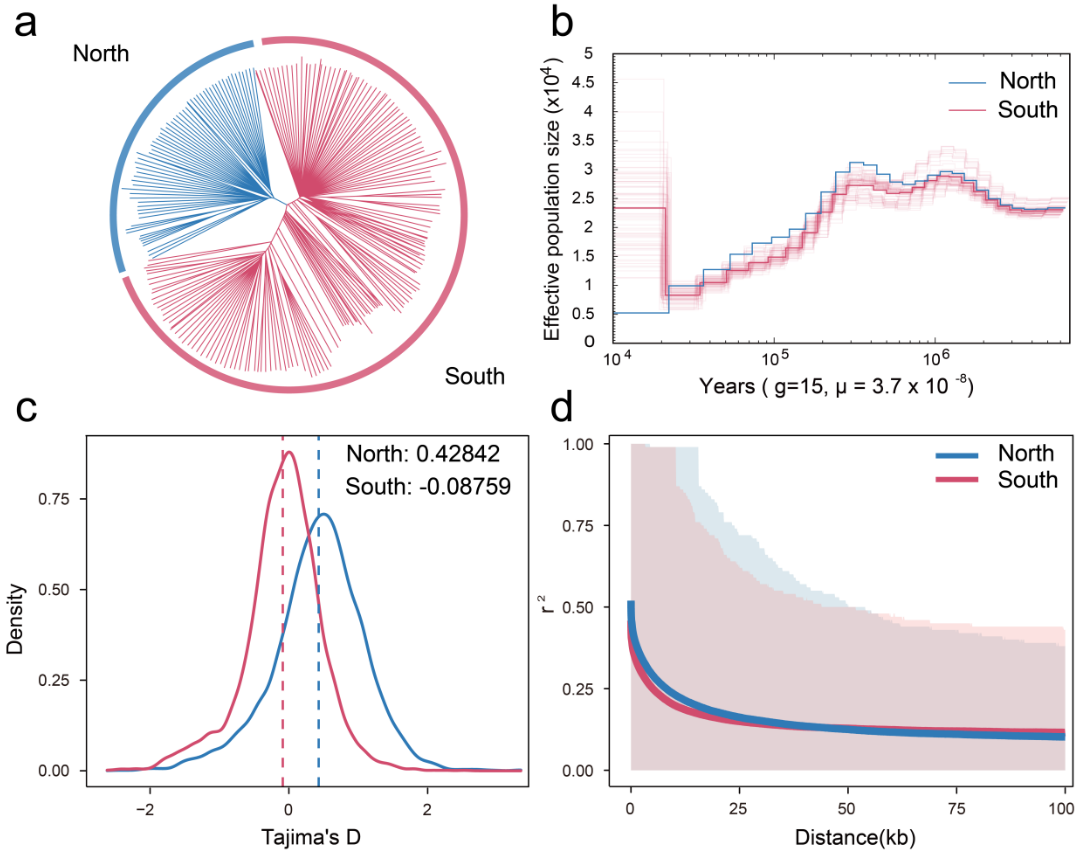

Supplementary Fig. 5. a, Neighbor-joining (NJ) phylogenetic tree of all 230 *P. koreana* individuals constructed based on genetic distance from whole-genome pruned SNPs. Two major clades (North and South) are indicated. **b,** Demographic history of south and north groups inferred by the PSMC (pairwise sequentially Markovian coalescent) model. Bold lines are the median estimates for the seven selected individuals from each of the two groups, whereas faint lines are 140 bootstrap replicates, with 10 replicates being conducted for each of the selected individuals from the two groups. **c,** The Tajima's D statistics of south and north groups over 100 Kbp non-overlapping windows across the genome. The dashed lines indicate the average estimates. **d,** Linkage disequilibrium (LD) decay estimated by PopLDdecay for two groups of *P. koreana* (thick lines) with the 90% ranges (shades; 5% to 95% percentiles) of r^2 values.

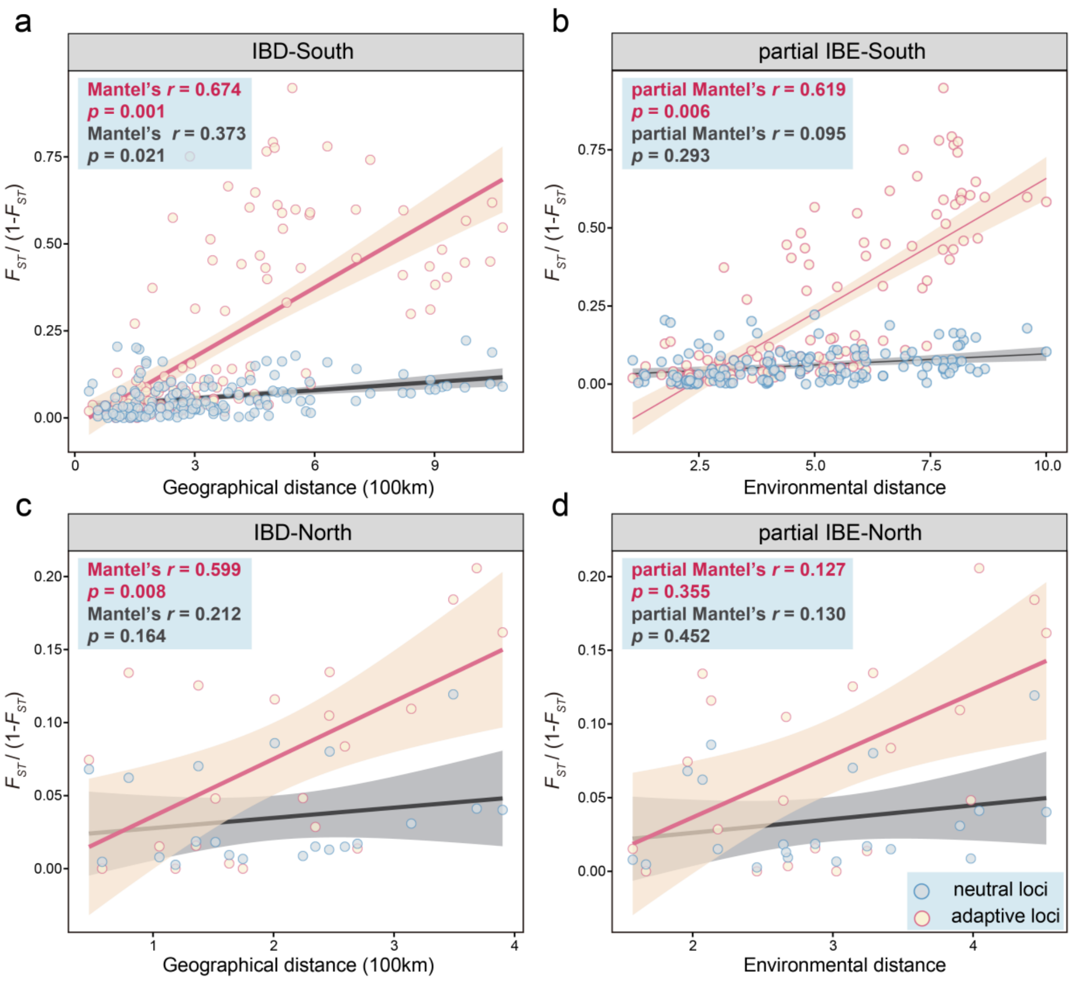

Supplementary Fig. 6. Isolation-by-distance (IBD) and Isolation-by-environment (IBE) analyses of potentially neutral and adaptive variants within southern and northern groups of populations. **a,c,** Mantel test (two-sided) for pairwise genetic distance $F_{ST}/(1-F_{ST})$ versus geographical distance for southern (n=136) and northern (n=21) populations, respectively. The orange and grey shadow of linear regressions denote the 95% confidence interval. **b,d,** Partial Mantel test (two-sided) for pairwise genetic distance $F_{ST}/(1-F_{ST})$ versus environmental distance after controlling for the effect of geographical distance for southern (n=136) and northern (n=21) populations, respectively. The orange and grey shadow of linear regressions denote the 95% confidence interval.

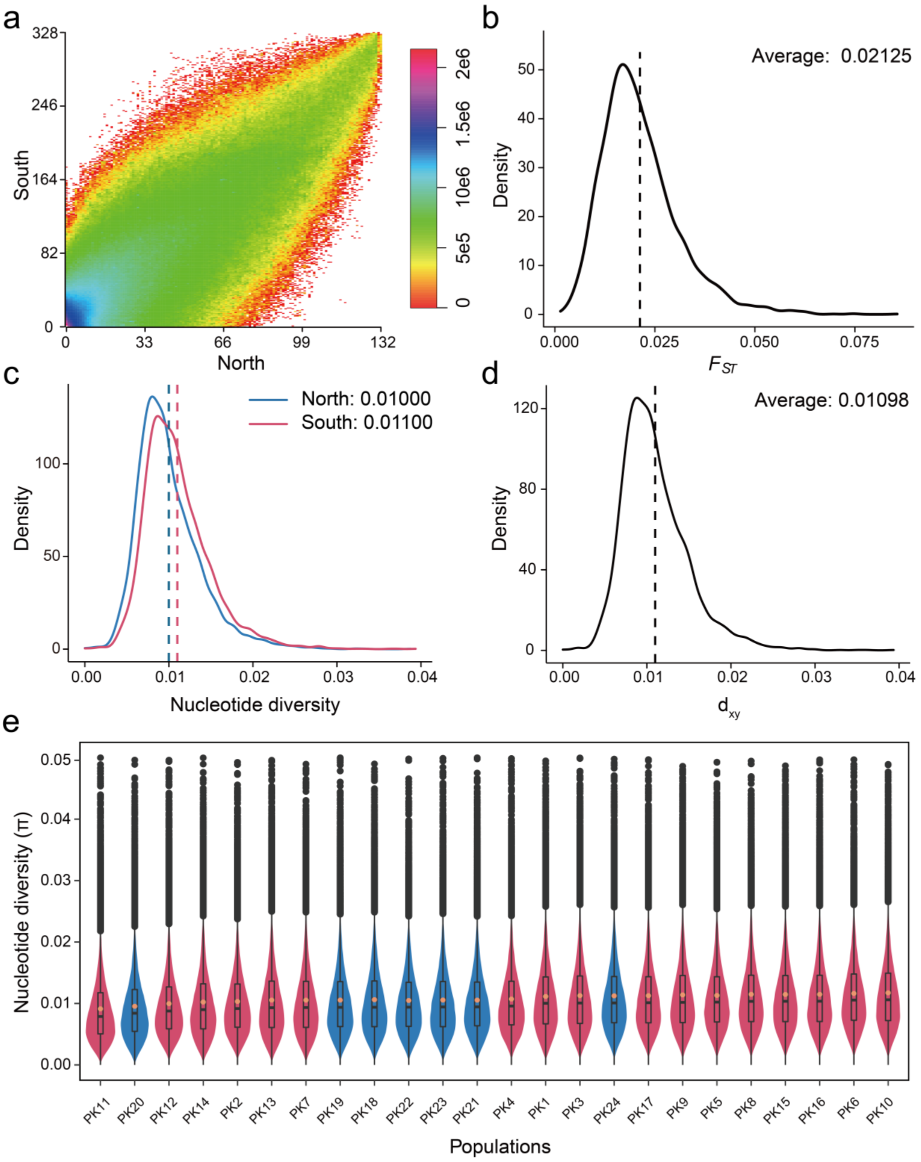

Supplementary Fig. 7. a, Two-dimensional site frequency spectrum of southern and northern groups of populations. **b,** The distribution of F_{ST} values between southern and northern groups of populations over 100 kbp non-overlapping windows across the genome. The dashed line indicates the average F_{ST} value. **c,** The nucleotide diversity (π) over 100 kbp non-overlapping windows across the genome for southern and northern groups of populations. **d,** The nucleotide divergence (d_{xy}) between southern and northern groups of populations over 100 kbp non-overlapping windows across the genome. The dashed lines indicate the average estimates. **e,** The distribution of nucleotide diversity over 10 kbp non-overlapping windows across the 24 *P. koreana* populations. The populations are arranged from low diversity to high (arranged by medians). The box plots show the mean values (orange points) and 25th−75th percentiles (box limits) of each population. The whiskers extend from the top/bottom to the maxima and minima. Data beyond the end of the whiskers are considered outliers.

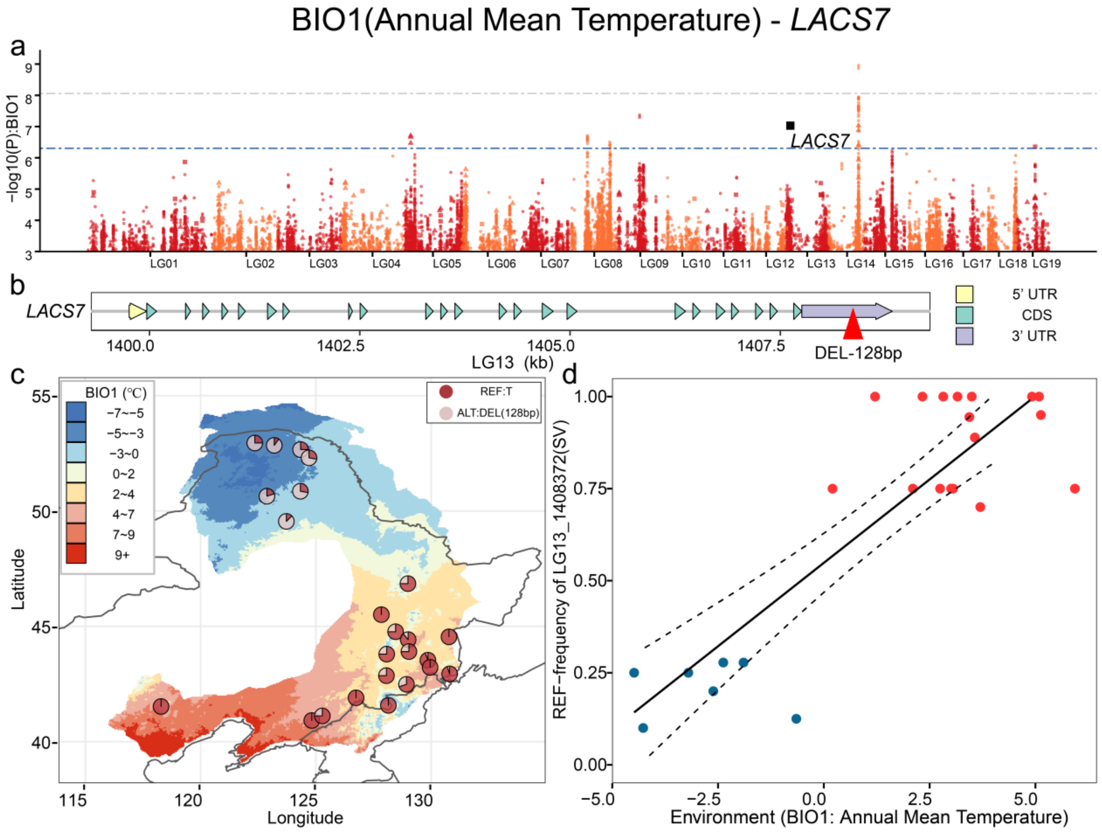

Supplementary Fig. 8. Genome-wide variation associated with each environmental variable (BIO5 and BIO13 are not included). a, Manhattan plot shows the genotype-environment association estimated with LFMM. SNPs, Indels and SVs are represented by points, triangles and squares, respectively. Two multiple comparisons methods were made: the blue or red dashed line represents the 5% false discovery rate correction thresholds; gray represents the Bonferroni correction, *P* value threshold of 0.05. Colors distinguish different chromosomes. **b,** Schematic diagram of the structure of the gene associated with the variant marked in **a**. **c,** Distribution of allele frequencies across 24 populations of the significant variant marked in **a**. Colors of raster on map represent the environmental variable under current scenario. **d,** Linear regression of allele frequencies and environment variables for the selected variant. Red and blue dots represent southern and northern populations, respectively. The dashed line represents the 95% confidence interval.

Continuation Supplementary Fig. 8.

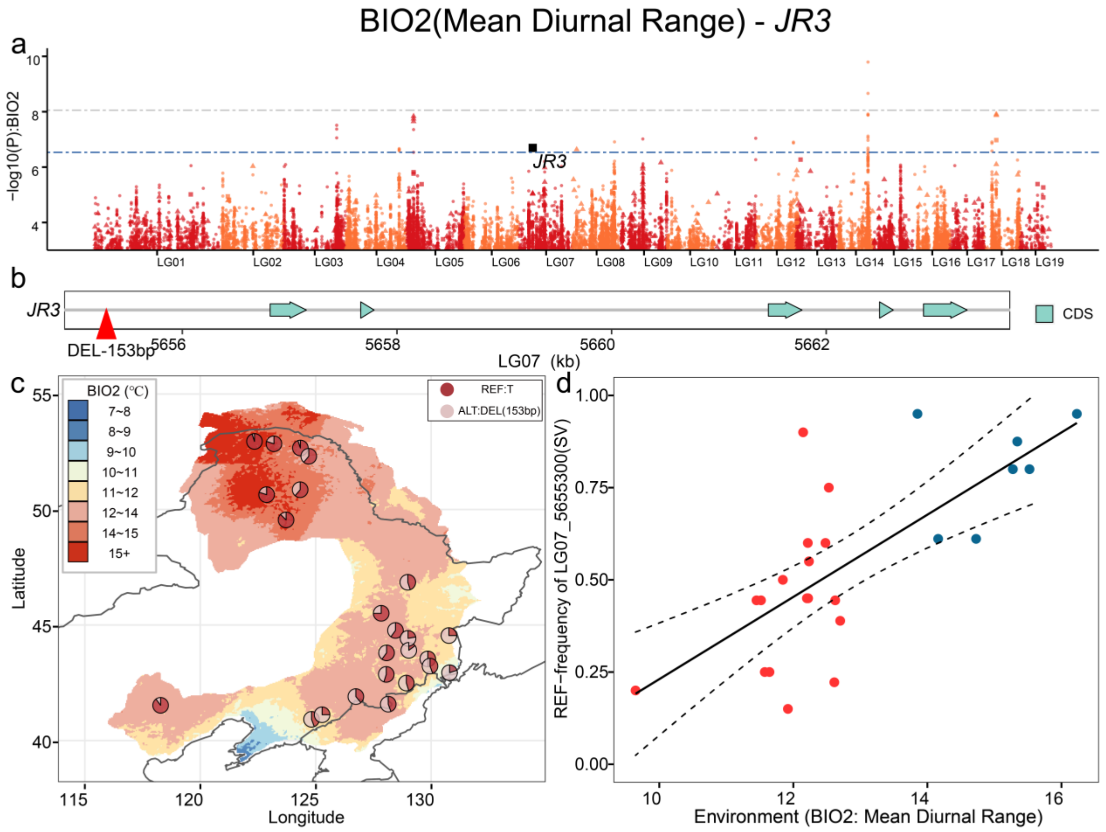

Continuation Supplementary Fig. 8.

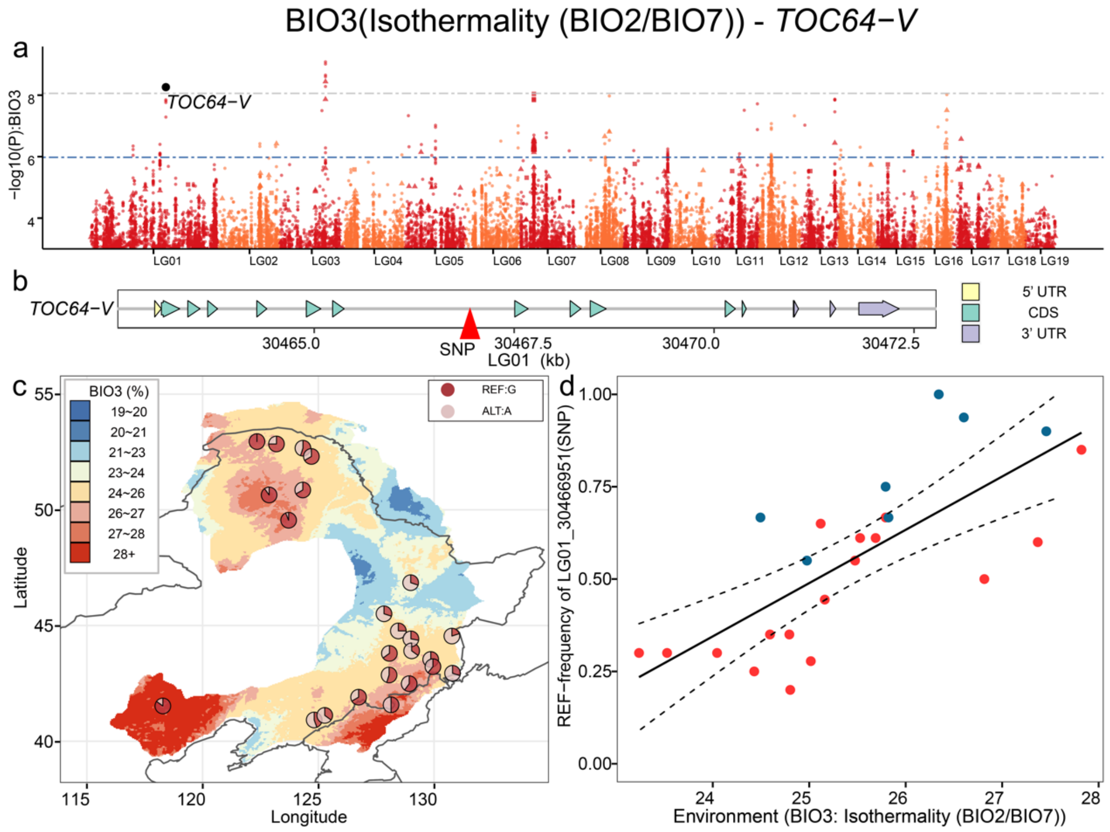

Continuation Supplementary Fig. 8.

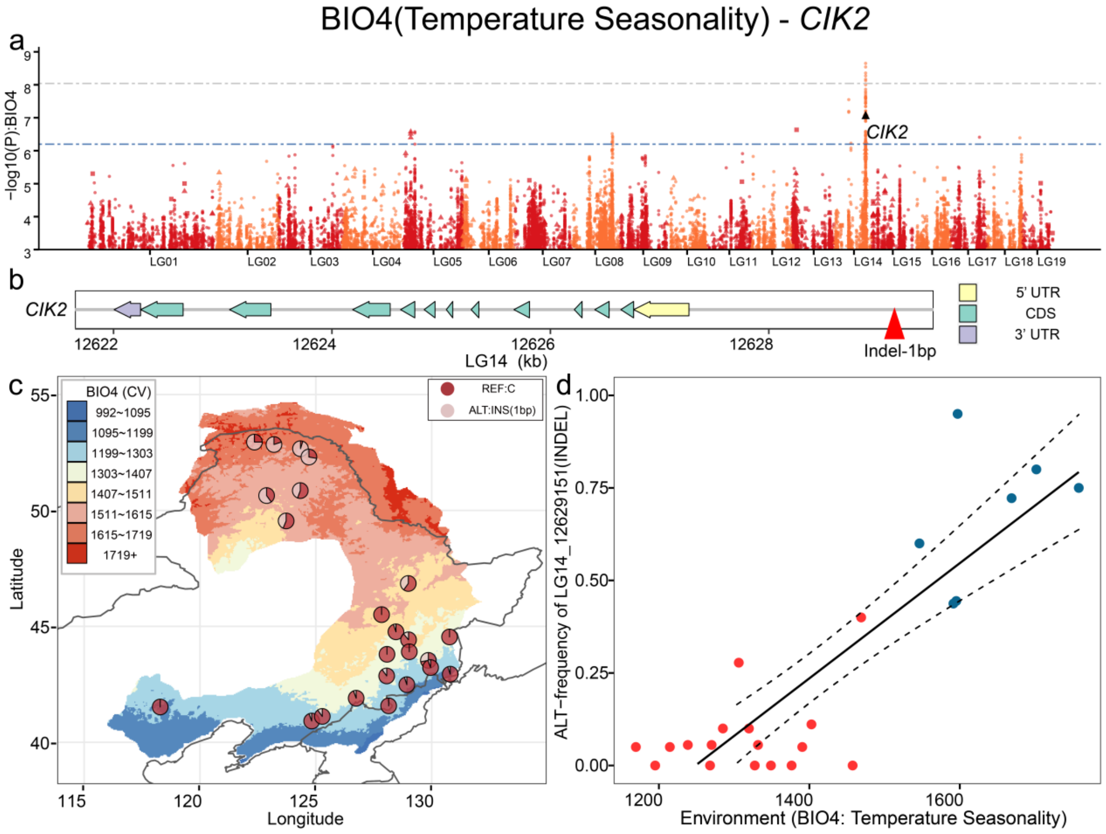

Continuation Supplementary Fig. 8.

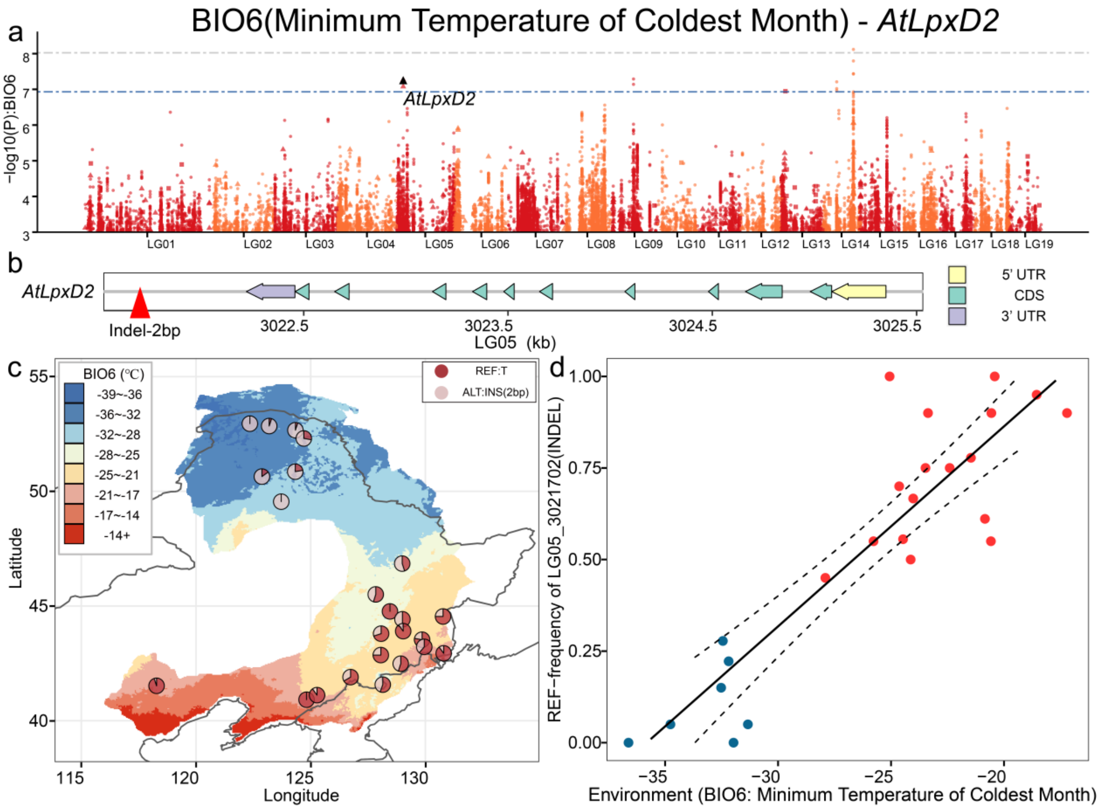

Continuation Supplementary Fig. 8.

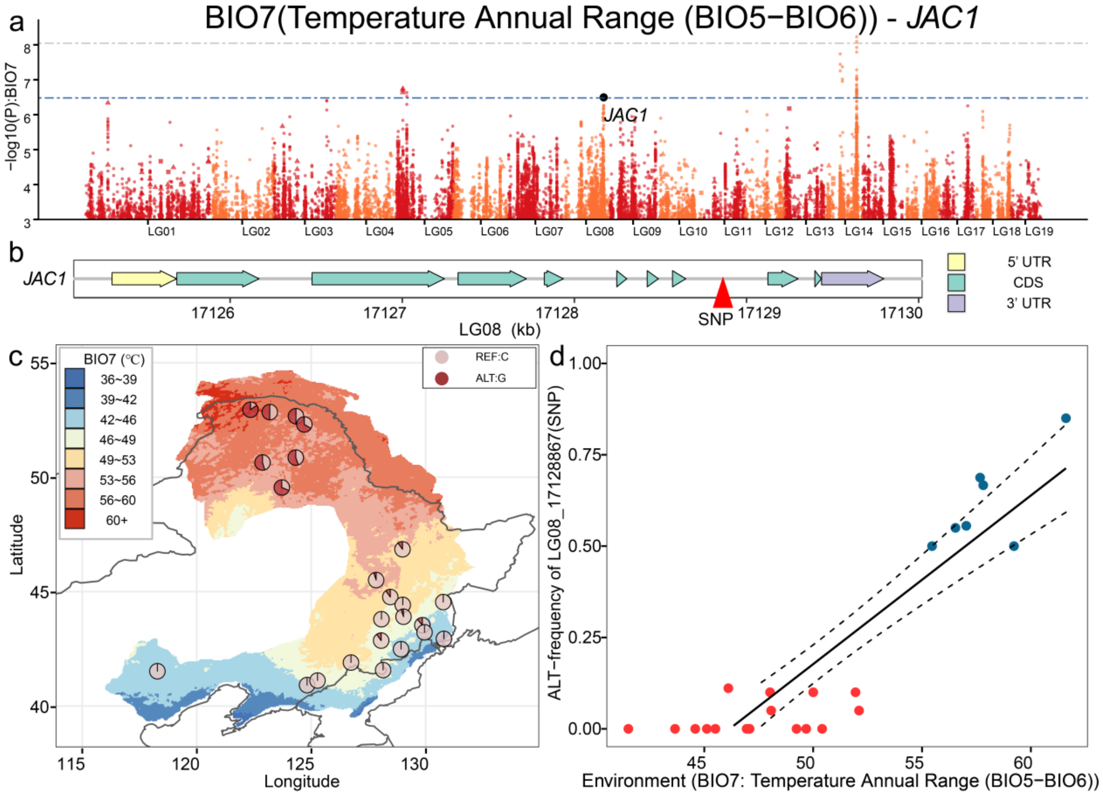

Continuation Supplementary Fig. 8.

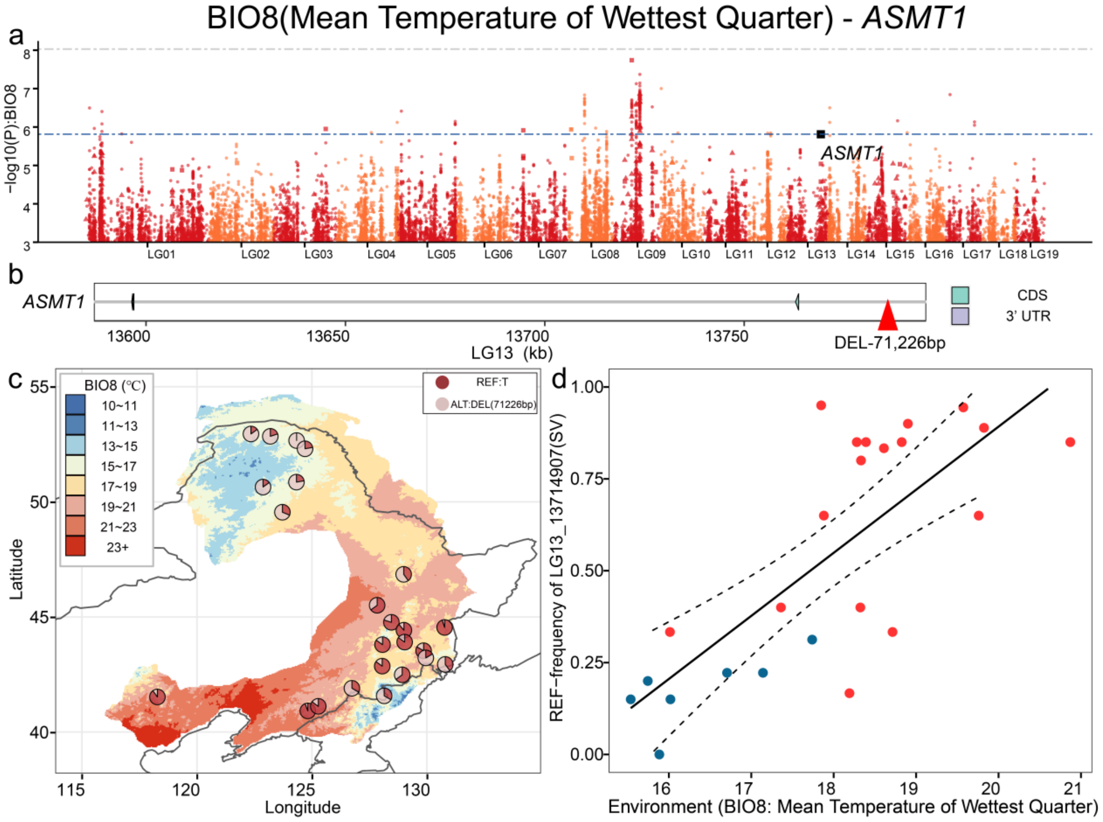

Continuation Supplementary Fig. 8.

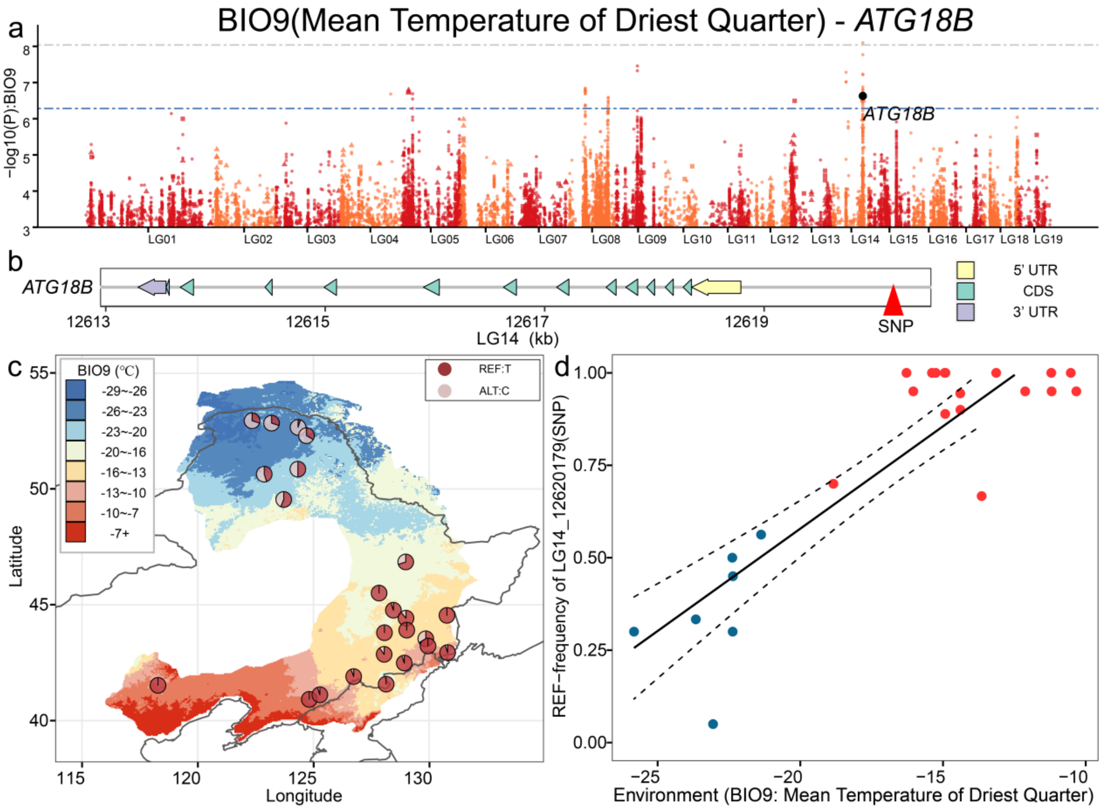

Continuation Supplementary Fig. 8.

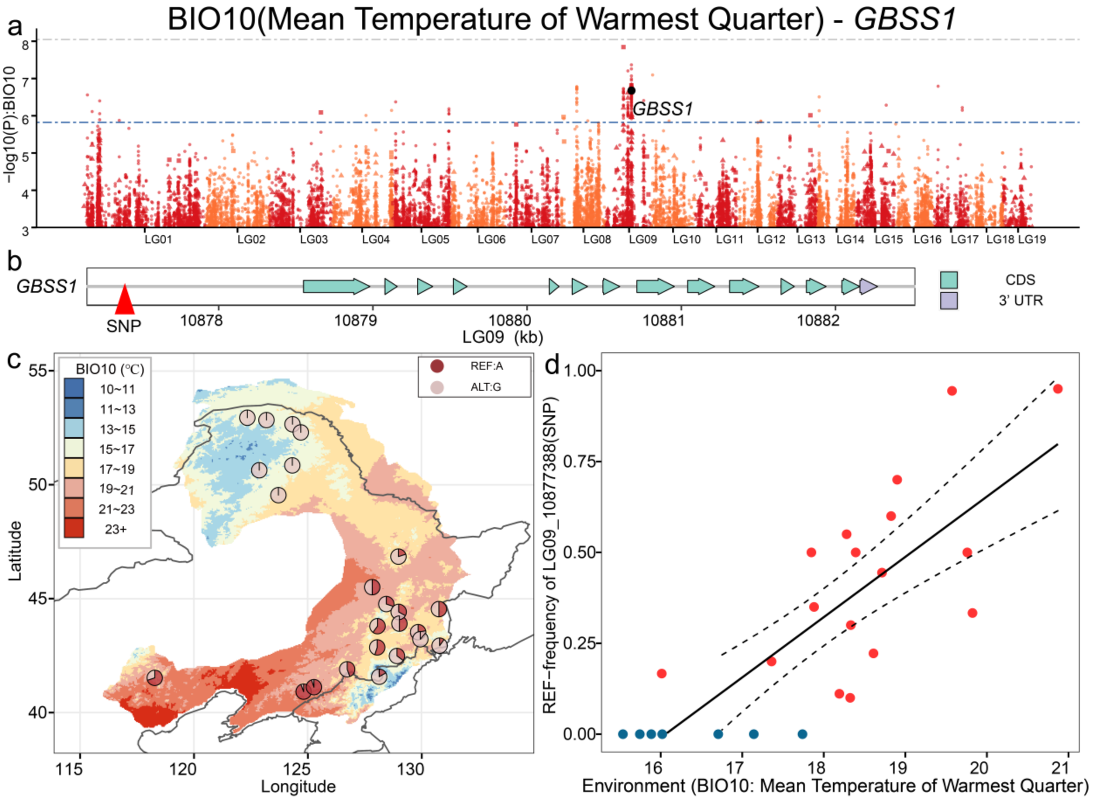

Continuation Supplementary Fig. 8.

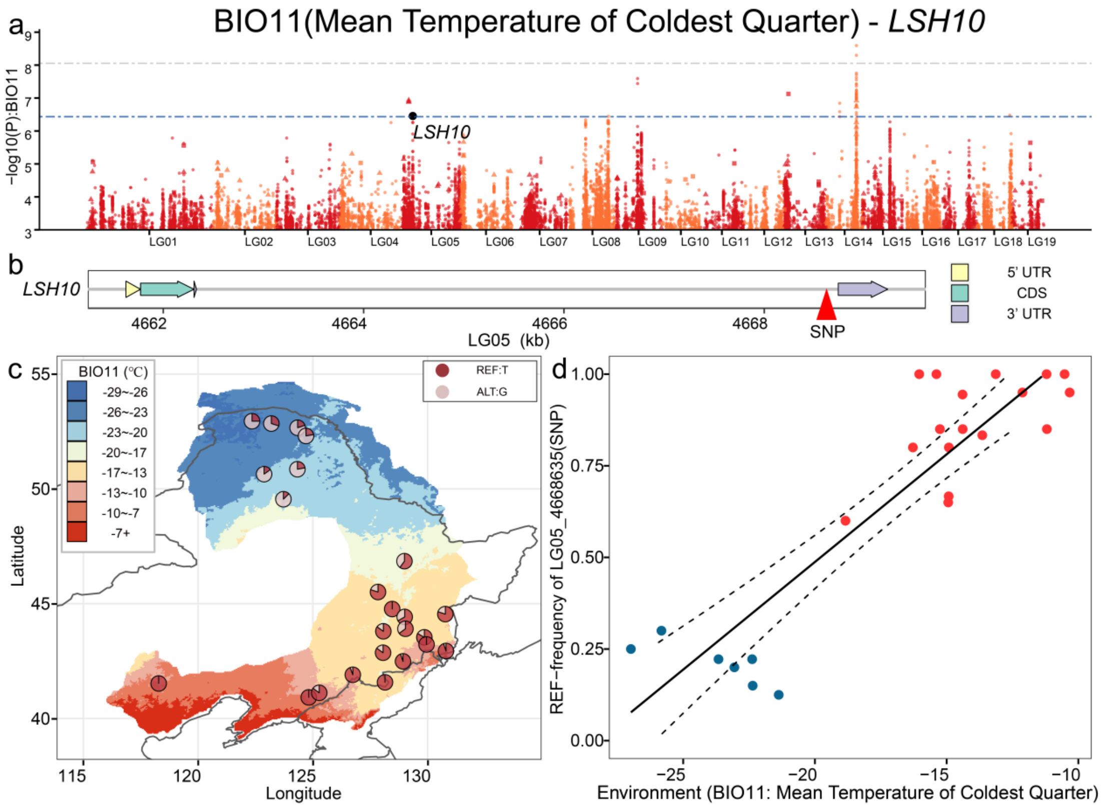

Continuation Supplementary Fig. 8.

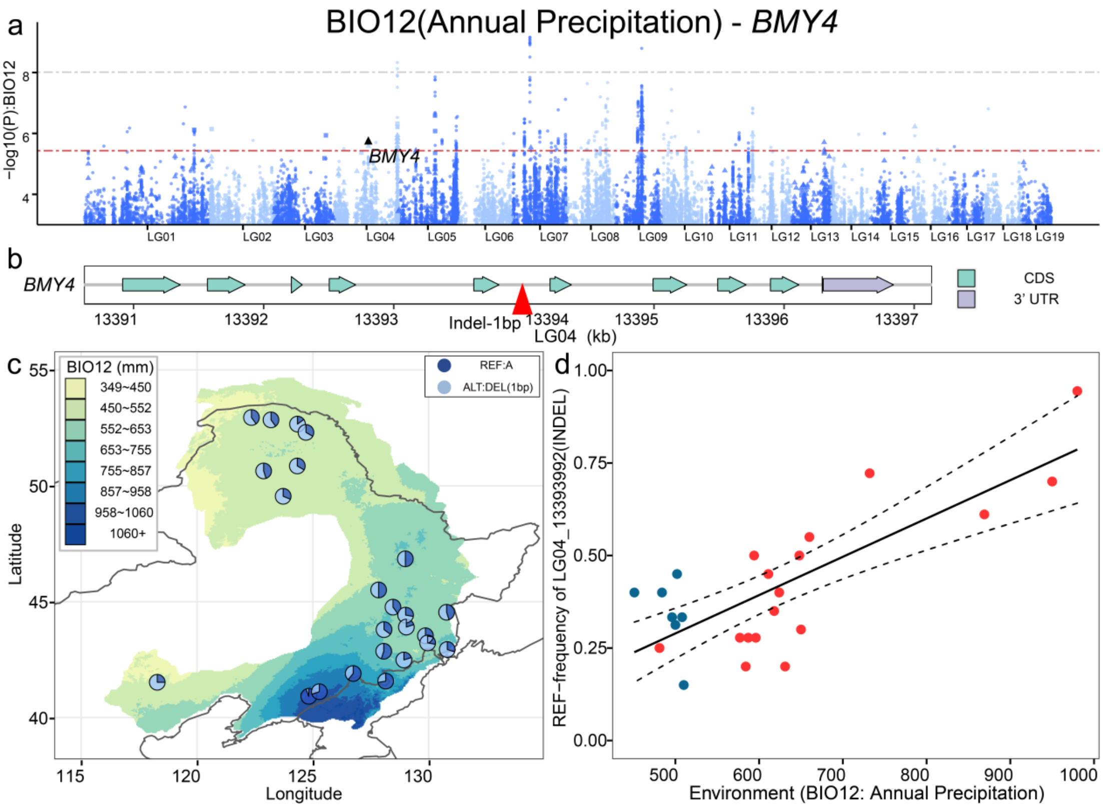

Continuation Supplementary Fig. 8.

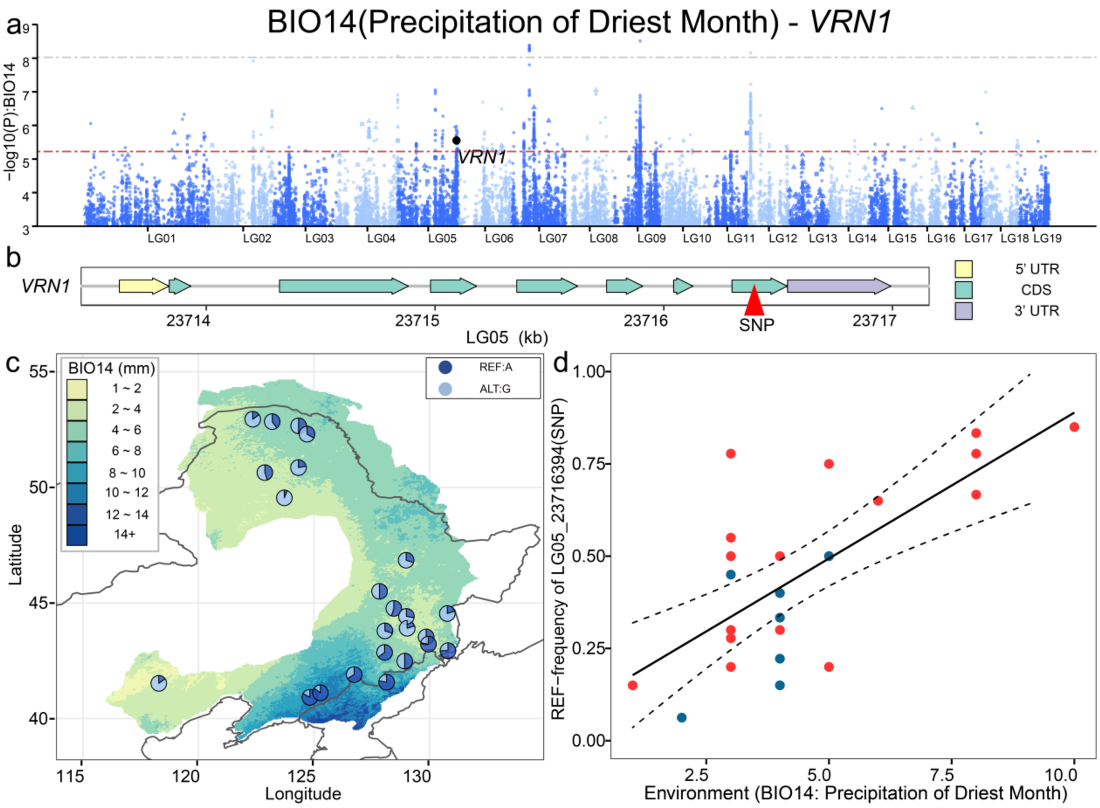

Continuation Supplementary Fig. 8.

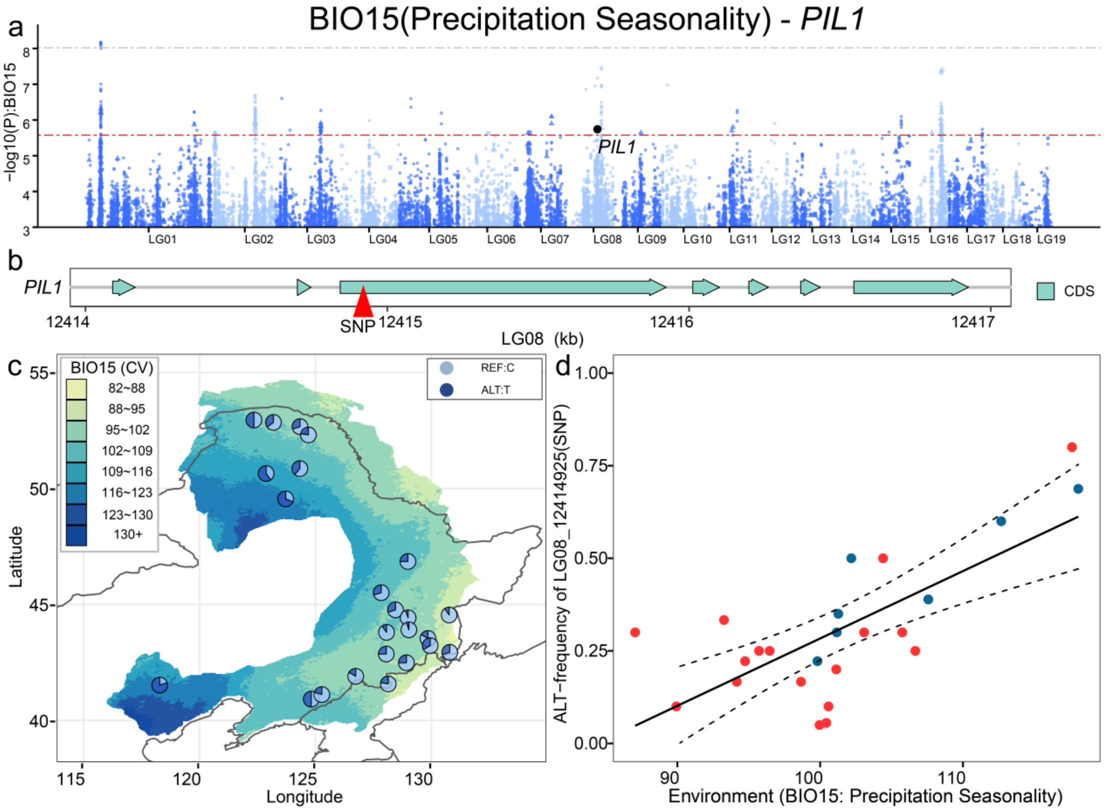

Continuation Supplementary Fig. 8.

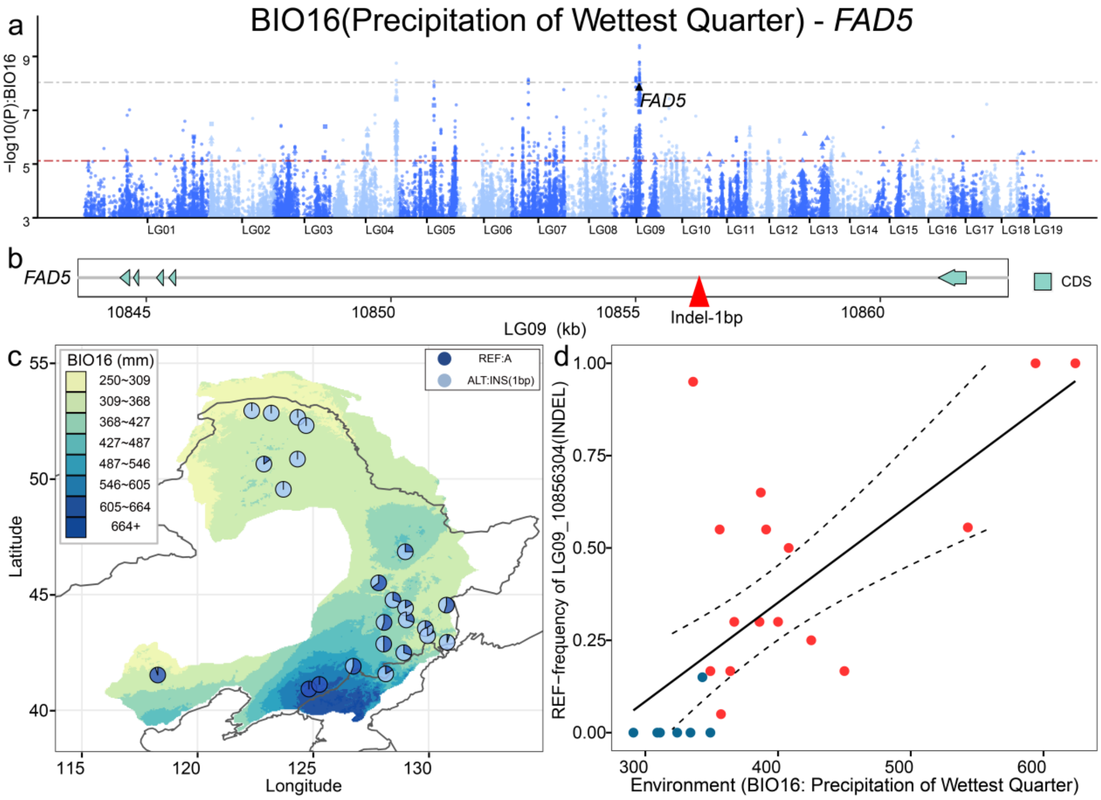

Continuation Supplementary Fig. 8.

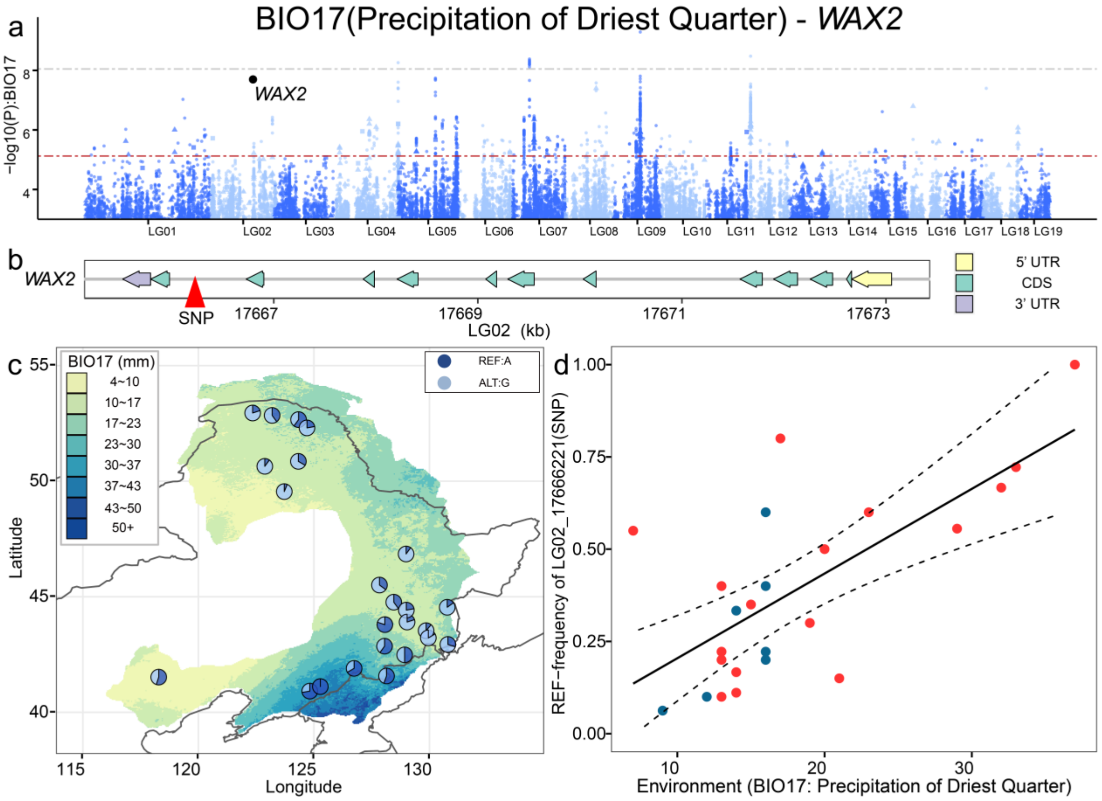

Continuation Supplementary Fig. 8.

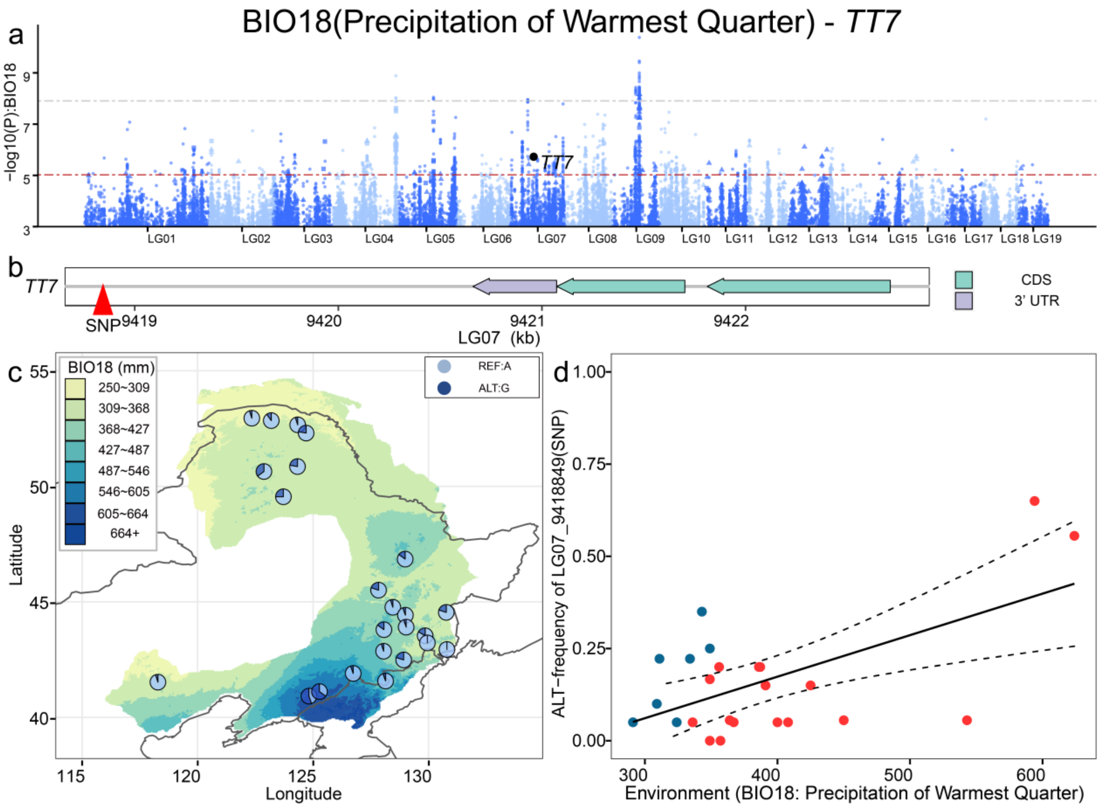

Continuation Supplementary Fig. 8.

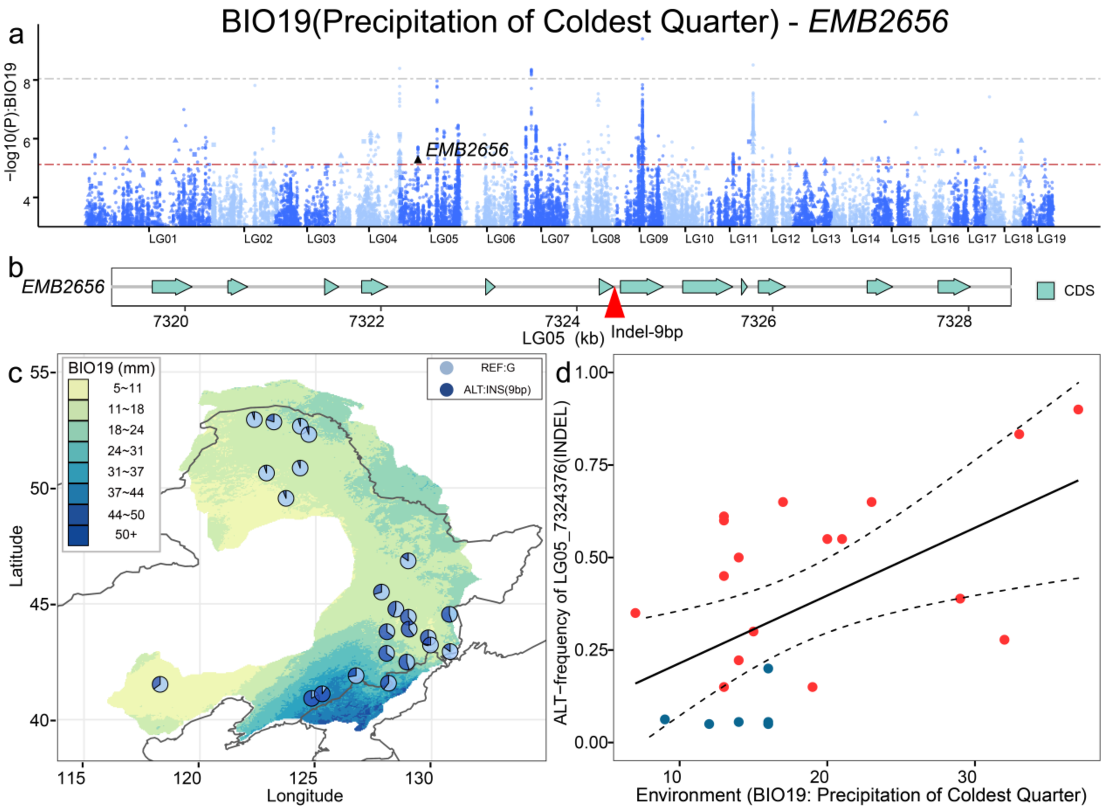

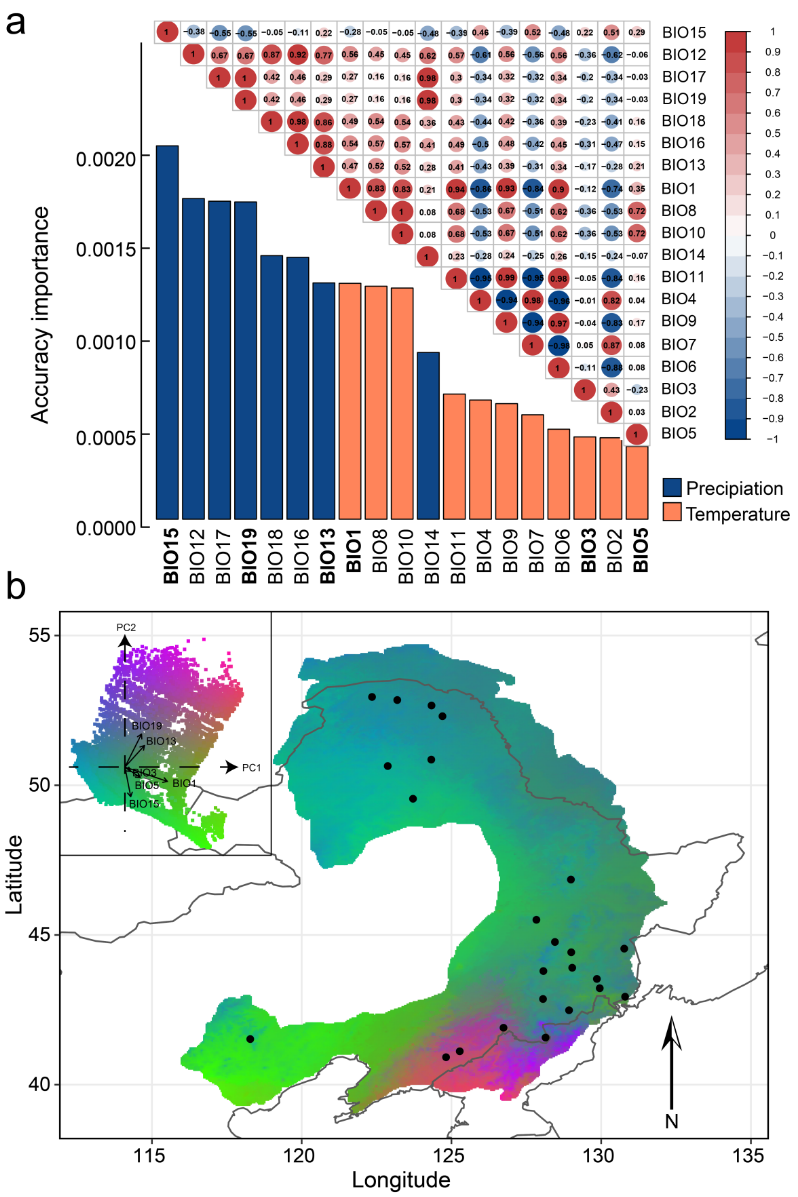

Supplementary Fig. 9. a, Spearman's correlation coefficient (two-sided test) of 19 environmental variables (above the diagonal), and ranked importance of 19 environmental variables based on gradient forest analysis (below the diagonal). The bold variables indicate the 6 environmental variables selected for RDA analysis. **b,** Gradient forest-transformed genotype-climate association across the distribution of *P. koreana* (n = 60,000 grids). Colors are based on the results of the principal components analysis (PCA) of transformed climate variables (six selected variables, bold in **a**), with the top three components transformed for visualization in a red-green-blue (RGB) color scale. Similar colors in the sampled space correspond to similar expected genetic composition at adaptive variants. Points on map represented 24 natural populations. Inset: The PCA-based biplot indicates the contribution of climate variables to the predicted pattern of genomic variation. Arrows show the loadings of the environmental variables.

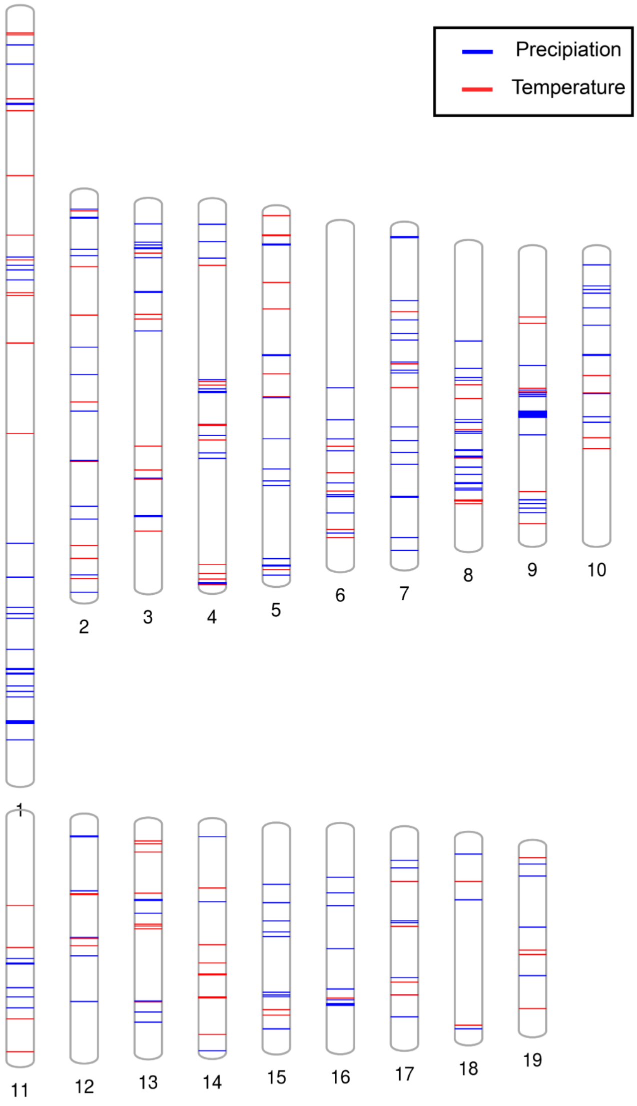

Supplementary Fig. 10. Mapping the location of the 1,779 core adaptive variants detected by both the approach of LFMM and RDA across the whole-genome. The blue and red bars indicate the adaptive variants associated with precipitation and temperature-related environmental variables, respectively.

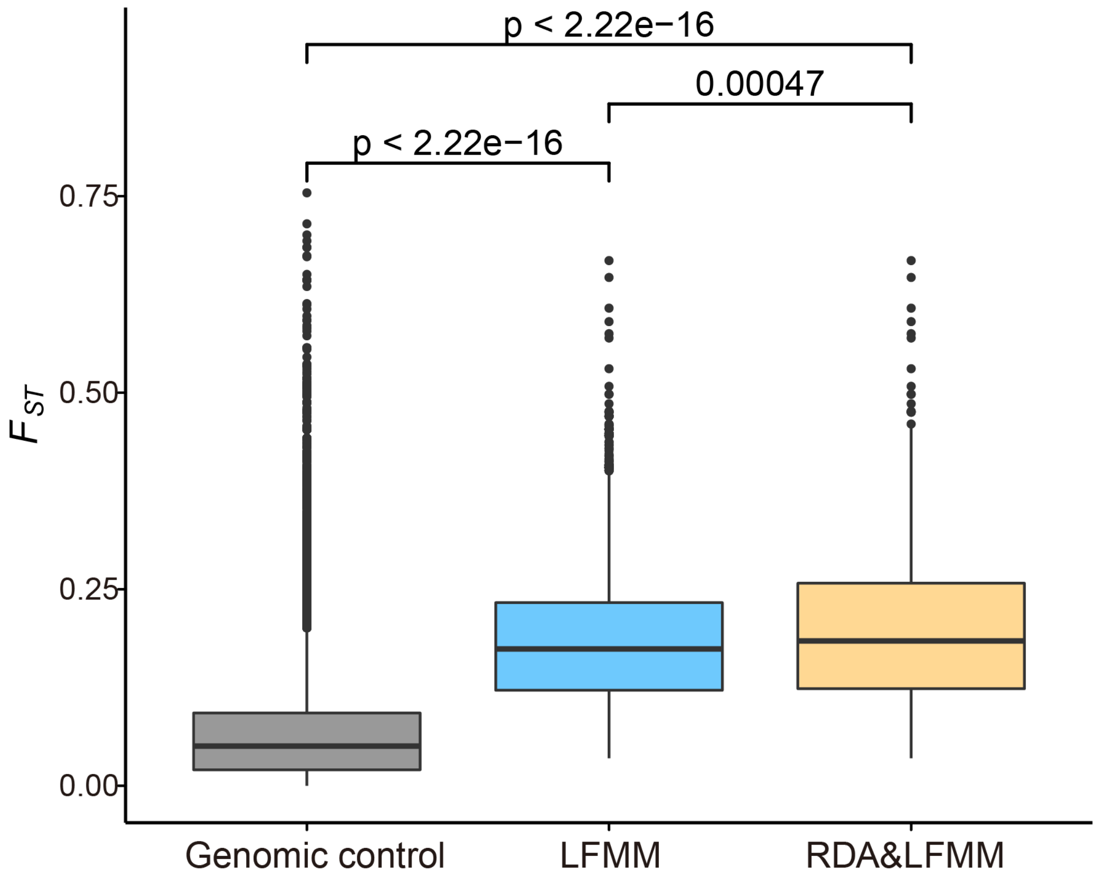

Supplementary Fig. 11. Comparison of genetic differentiation (F_{ST}) for group pair (south and north) calculated by using 100,000 random variants (Genomic control), 3,435 environmental-associated variants detected by LFMM (LFMM) and 1,779 core adaptive variants detected by both LFMM and RDA (RDA&LFMM), respectively. The box plots show the median (centre line) and 25th−75th percentiles (box limits). The whiskers extend from the top/bottom to the maxima and minima. Data beyond the end of the whiskers are considered outliers. The Wilcoxon test (two-tailed) was used to determine the significance.

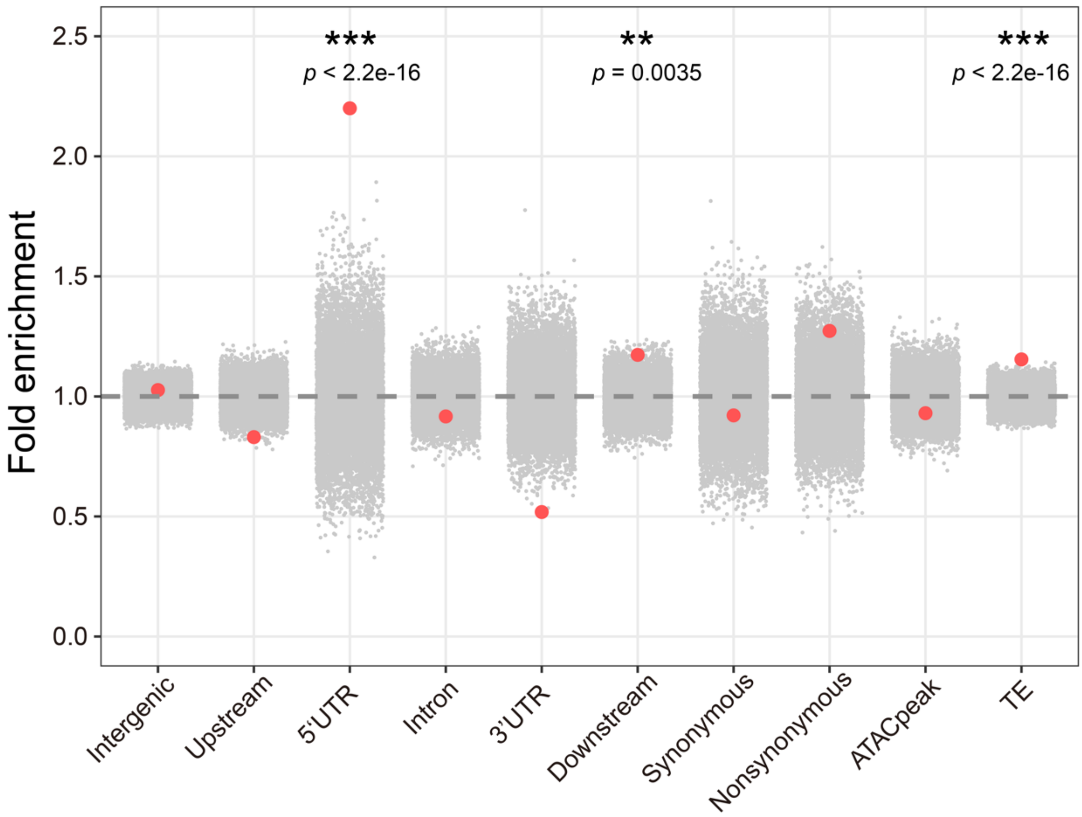

Supplementary Fig. 12. Enrichment of various functional categories in 1,779 core adaptive variants detected by both LFMM and RDA (red points). Grey dots show the distribution of results with 10,000 bootstrap replicates. The dashed line shows the expected enrichment under the null hypothesis of no enrichment. The significance is assessed by permutation test, with the significant enrichment being denoted by asterisks (*** $P < 0.001$, ** $P < 0.01$, * $P < 0.05$, ^{ns} $P > 0.05$).

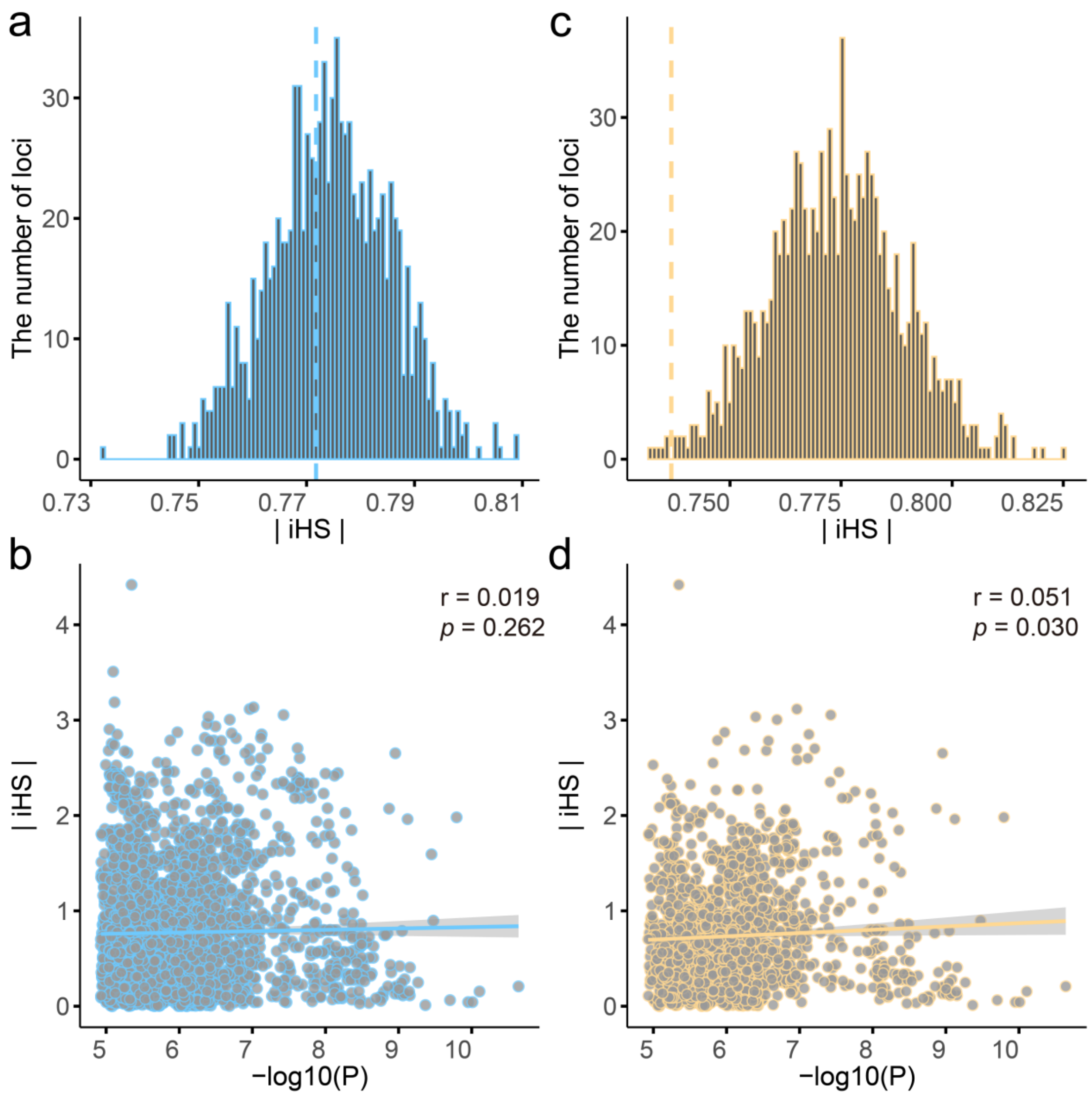

Supplementary Fig. 13. Weak selection signals for the environmental-associated variants. **a,c,** The observed average values of |iHS| scores (dashed lines) relative to the genome-wide distribution (derived from 1,000 bootstrap replicates) for the 3,435 variants detected by LFMM (**a**, blue lines) and 1,779 adaptive variants detected by both LFMM and RDA methods (**c**, yellow lines). **b,d,** No significant relationship between signals of selection (|iHS|) and environmental associations (-log10P) for the 3,435 variants detected by LFMM (**b**, blue dots) or 1,779 adaptive variants detected by both LFMM and RDA methods (**d**, yellow dots). The significance is determined by two-sided Spearman test. The shade represents the 95% confidence interval.

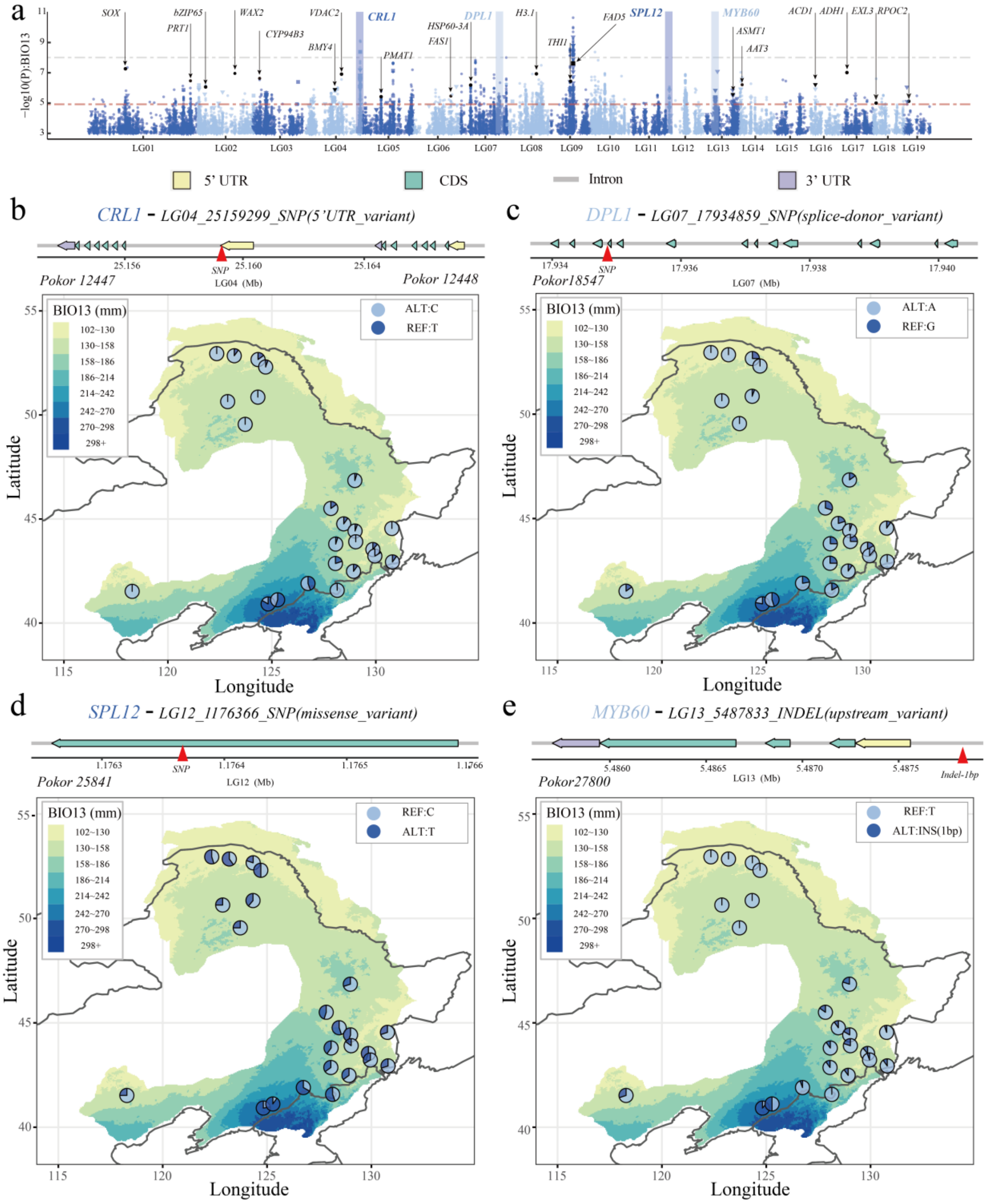

Supplementary Fig. 14. Display of some well-studied genes significantly related to BIO13 (precipitation of the wettest month). a, Manhattan plot shows the genotype-environment association estimated with LFMM. SNPs, Indels and SVs are represented by points, triangles and squares, respectively. Two multiple comparisons methods were made: the red dashed line represents the 5% false discovery rate correction threshold; gray represents the Bonferroni correction, adjusted P = 0.05. Colors distinguish different chromosomes. **b-e,** The gene structure of selected genes, marked in **a** (upper panels). The distribution of allele frequencies of an example variation of corresponding gene across 24 populations (the files of gene structure and population allele frequencies are provided in the source data). Colors of raster on map represent the environmental variable under current scenario.

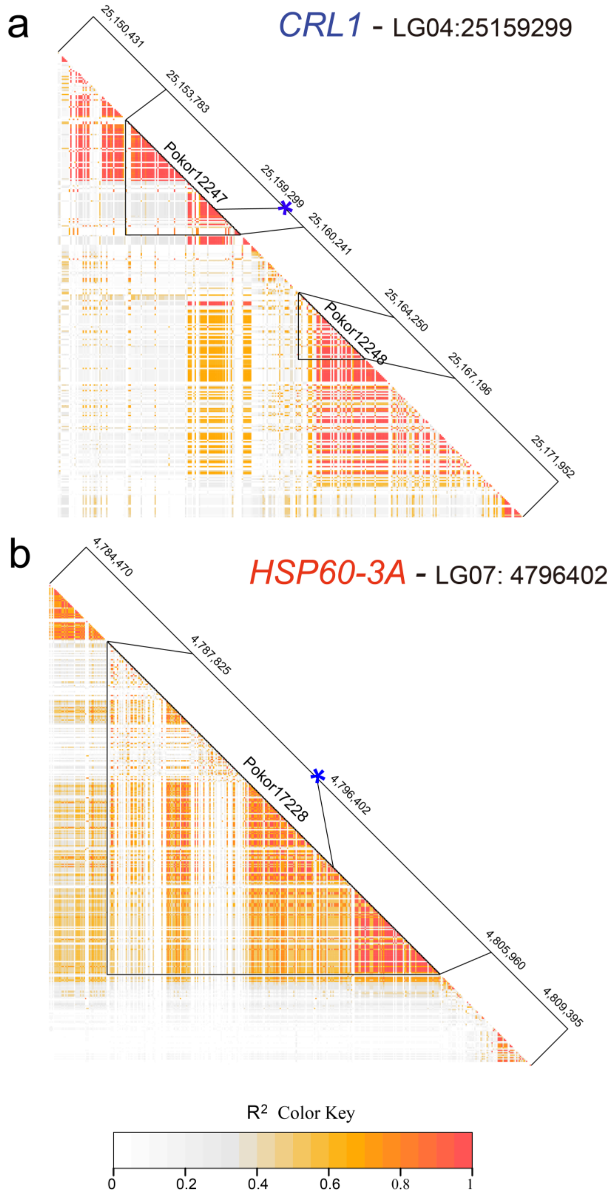

Supplementary Fig. 15. Heatmap of linkage disequilibrium surrounding the two candidate genes shown in **Fig. 3** (**a** *CRL1*; **b** *HSP60-3A*). The stars indicate the two representative SNPs (LG04:25159299 and LG07:4796402 for *CRL1* and *HSP60-3A*, respectively) shown in **Fig. 3**.

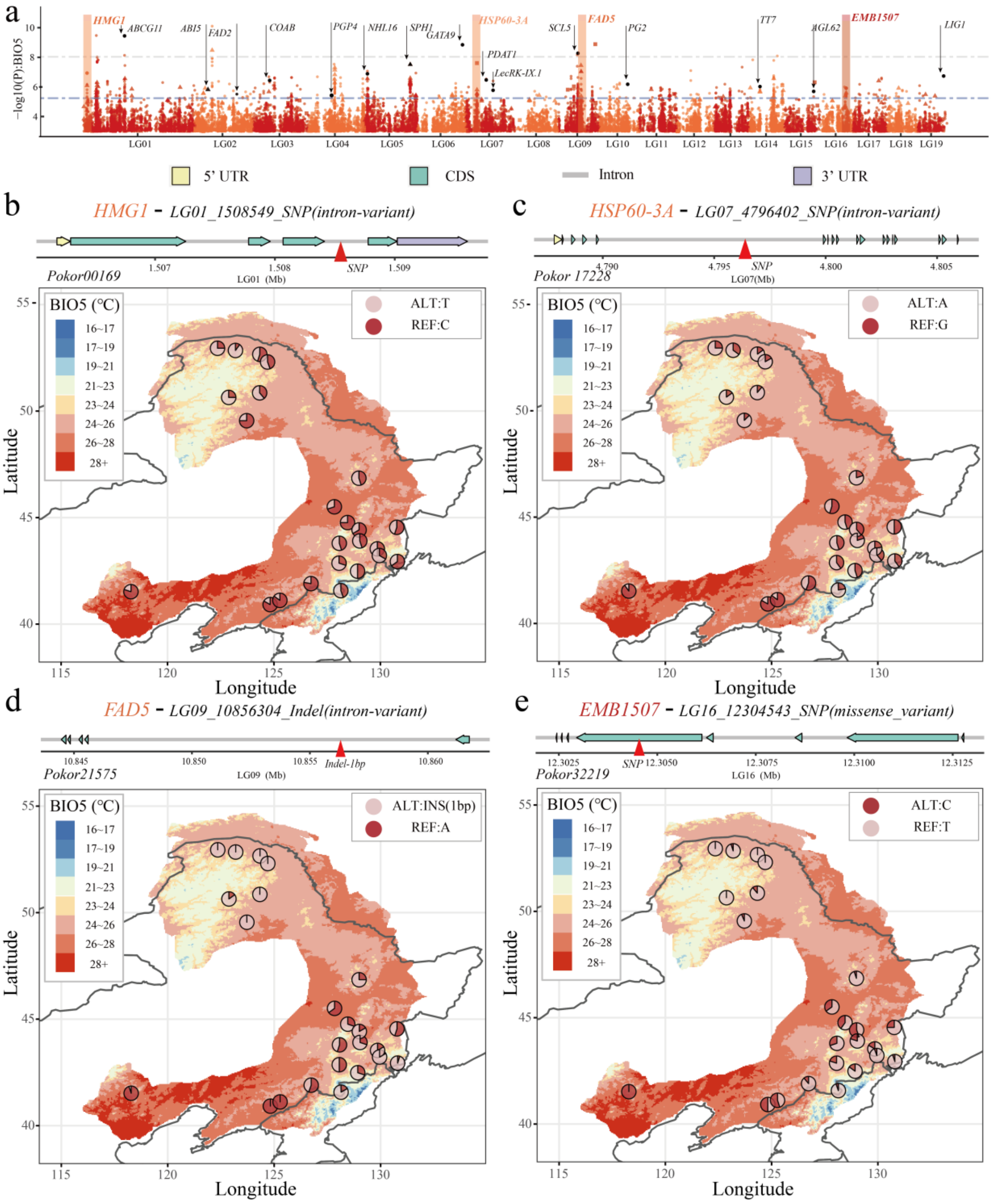

Supplementary Fig. 16. Display of some well-studied genes significantly related to BIO5 (maximum temperature of warmest month). a, Manhattan plot shows the genotype-environment association estimated with LFMM. SNPs, Indels and SVs are represented by points, triangles and squares, respectively. Two multiple comparisons methods were made: the blue dashed line represents the 5% false discovery rate correction threshold; gray represents the Bonferroni correction, *P* value threshold of 0.05. Colors distinguish different chromosomes. **b-e,** The gene structure of selected genes, marked in **a** (upper panels). The distribution of allele frequencies of an example variation of corresponding gene across 24 populations (the files of gene structure and population allele frequencies are provided in the source data). Colors of raster on map represent the environmental variable under current scenario.

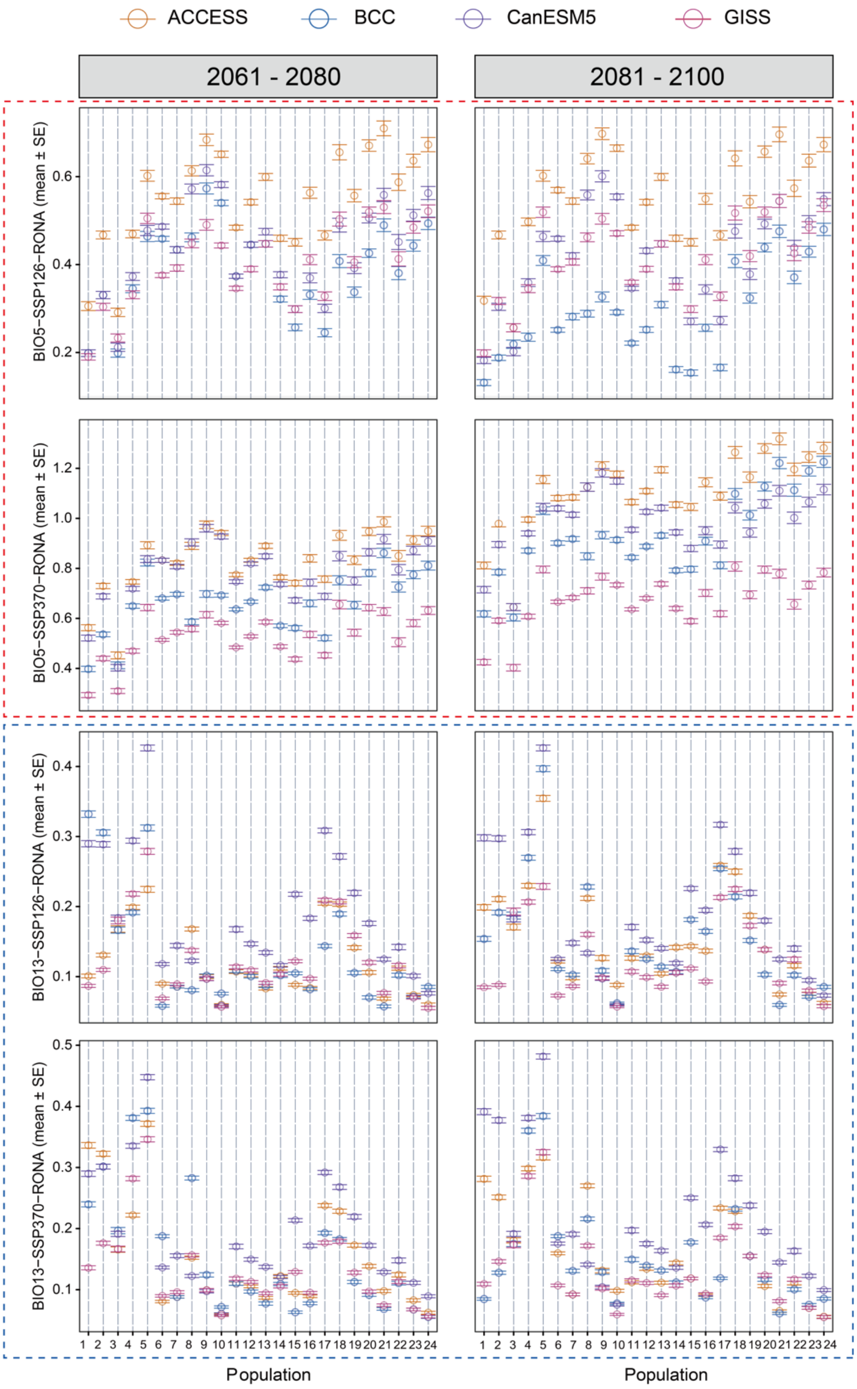

Supplementary Fig. 17. Comparison of risk of non-adaptedness (RONA) estimates across the 24 populations using the four different future climate models (represented by different colors) for BIO5 (upper four marked by red dashed line, 271 climatic-associate variants) and BIO13 (lower four marked by blue dashed line, 841 climatic-associate variants) under two scenarios of shared socioeconomic pathways SSP126 and SSP370 in 2061-2080 and 2081-2100, respectively. Error bars represent standard error of the average RONA (SE) for a given model.

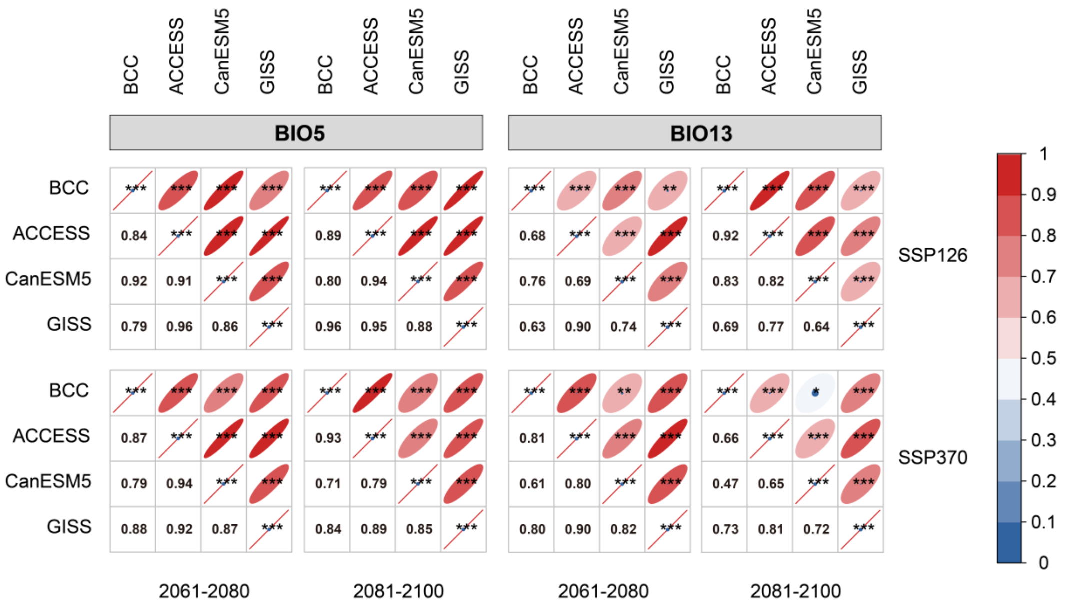

Supplementary Fig. 18. The pairwise correlations of the risk of non-adaptedness (RONA) values estimated among four future climate models. The significance was determined using two-tailed Spearman's correlation analysis. Asterisks indicate significance levels (*P < 0.05, **P< 0.01, ***P<0.001). BCC: BCC-CSM2-MR; ACCESS: ACCESS-CM2; GISS: GISS-E2-1-G.

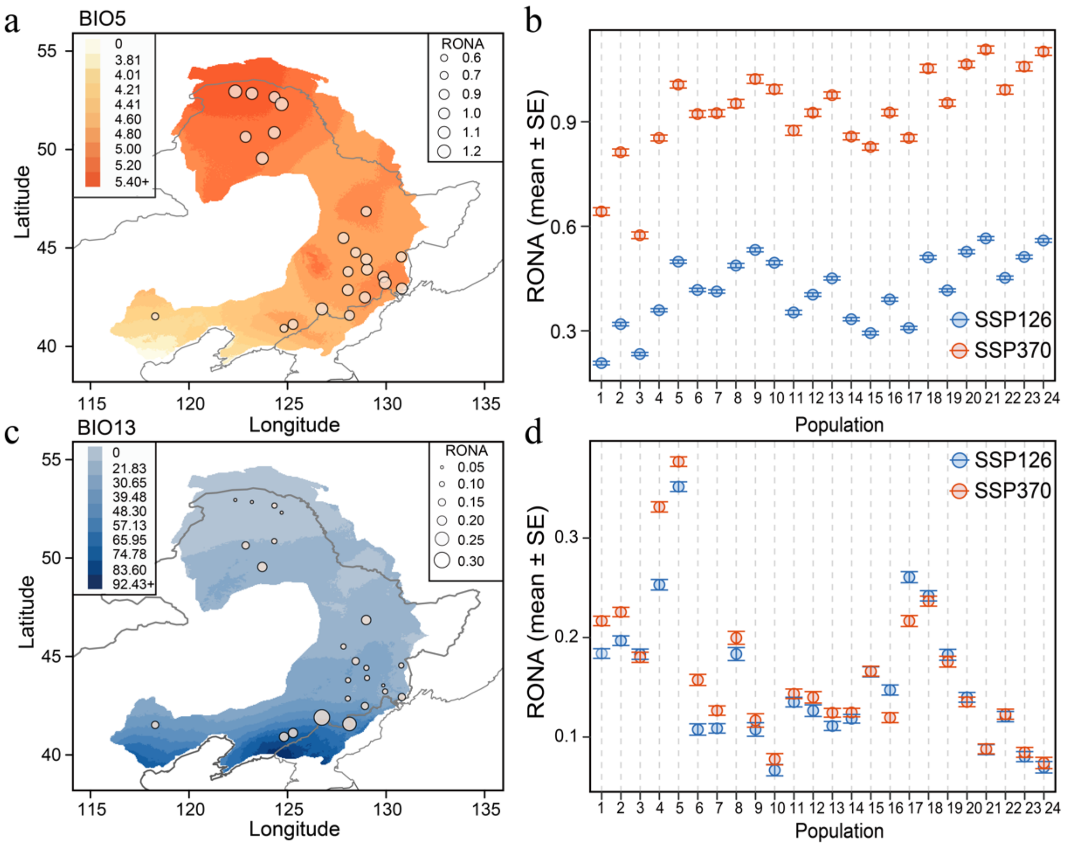

Supplementary Fig. 19. Risk of non-adaptedness (RONA) of *P. koreana* to future climatic conditions**. a,c,** The average RONA estimates across four climate models for the 24 populations under the SSP370 climate scenarios in 2081-2100 (**a** BIO5; **c** BIO13) . The raster colors on the map represent the degree of projected future climate change (absolute change). Areas with darker red (**a**) or blue (**c**) are predicted to experience more dramatic change in the respective climate variables. The size of circles on the map represents RONA values of different natural populations. **b,d,** Comparison of the average RONA values under two different climate scenarios (SSP 126 and SS370) in 2081-2100 across populations for BIO5 (**b** 271 climatic-associate variants) and BIO13 (**d** 841 climatic-associate variants), respectively. Error bars represent standard error of the average RONA (SE).

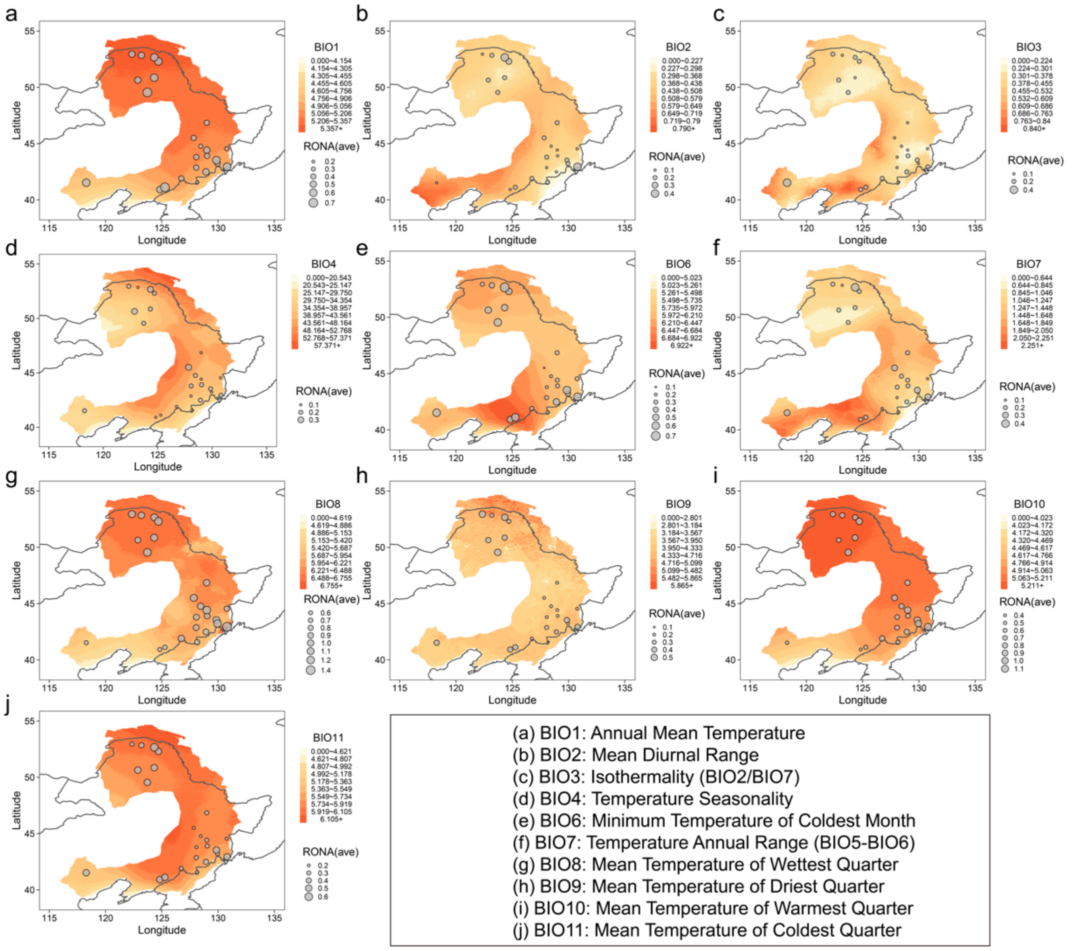

Supplementary Fig. 20. a-j Comparison of the average RONA estimates across four climate models of 24 *P. koreana* populations for temperature-related variables under SSP370 in 2061-2080, including BIO1 (**a**), BIO2 (**b**), BIO3 (**c**), BIO4 (**d**), BIO6 (**e**), BIO7 (**f**), BIO8 (**g**), BIO9 (**h**), BIO10 (**i**) and BIO11 (**j**). The raster colors on the maps represent the degree of projected future climate change (absolute change; the average of 4 climate models of SSP370 in 2061-2080). Areas with darker red are predicted to experience more dramatic change in the respective climate variables. The size of circles on the map represents RONA values of different natural populations. The abbreviation and full name of each environment variable are shown in the lower box.

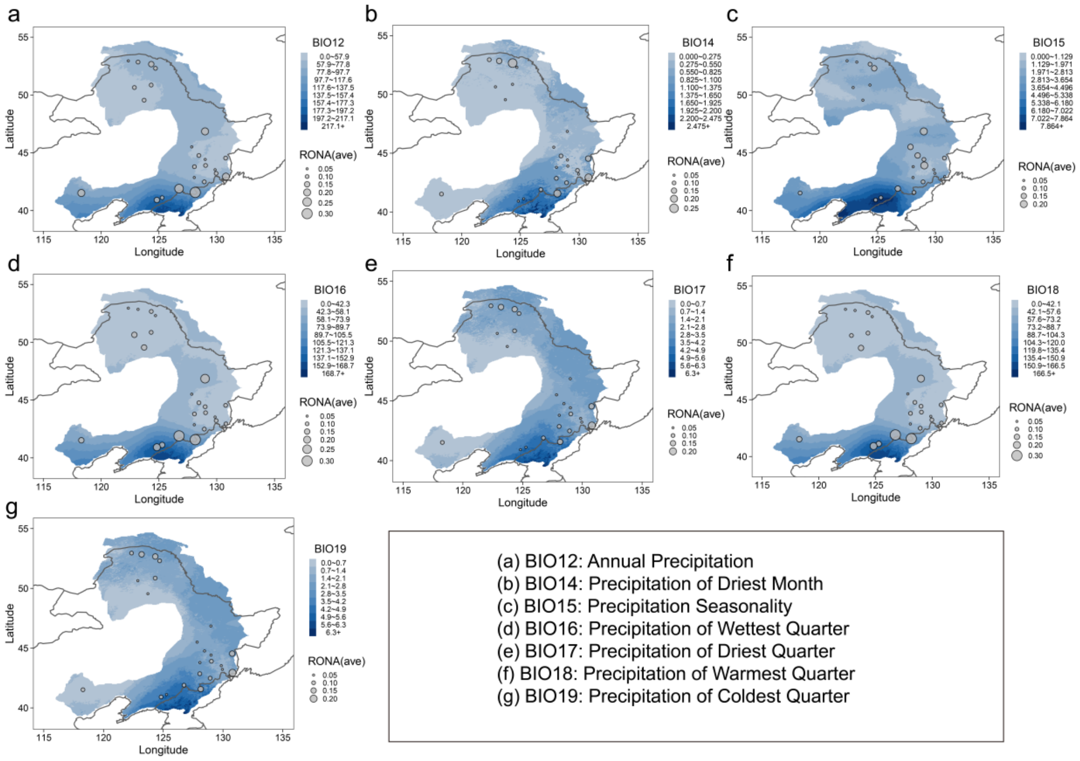

Supplementary Fig. 21. a-g Comparison of the average RONA estimates across four climate models of 24 *P. koreana* populations for precipitation-related variables under SSP370 in 2061-2080, including BIO12 (**a**), BIO14 (**b**), BIO15 (**c**), BIO16 (**d**), BIO17 (**e**), BIO18 (**f**) and BIO19 (**g**). The raster colors on the map represent the degree of projected future climate change (absolute change; the average of 4 climate models of SSP370 in 2061-2080). Areas with darker blue are predicted to experience more dramatic change in the respective climate variables. The size of circles on the map represents RONA values of different natural populations. The abbreviation and full name of each environment variable are shown in the lower box.

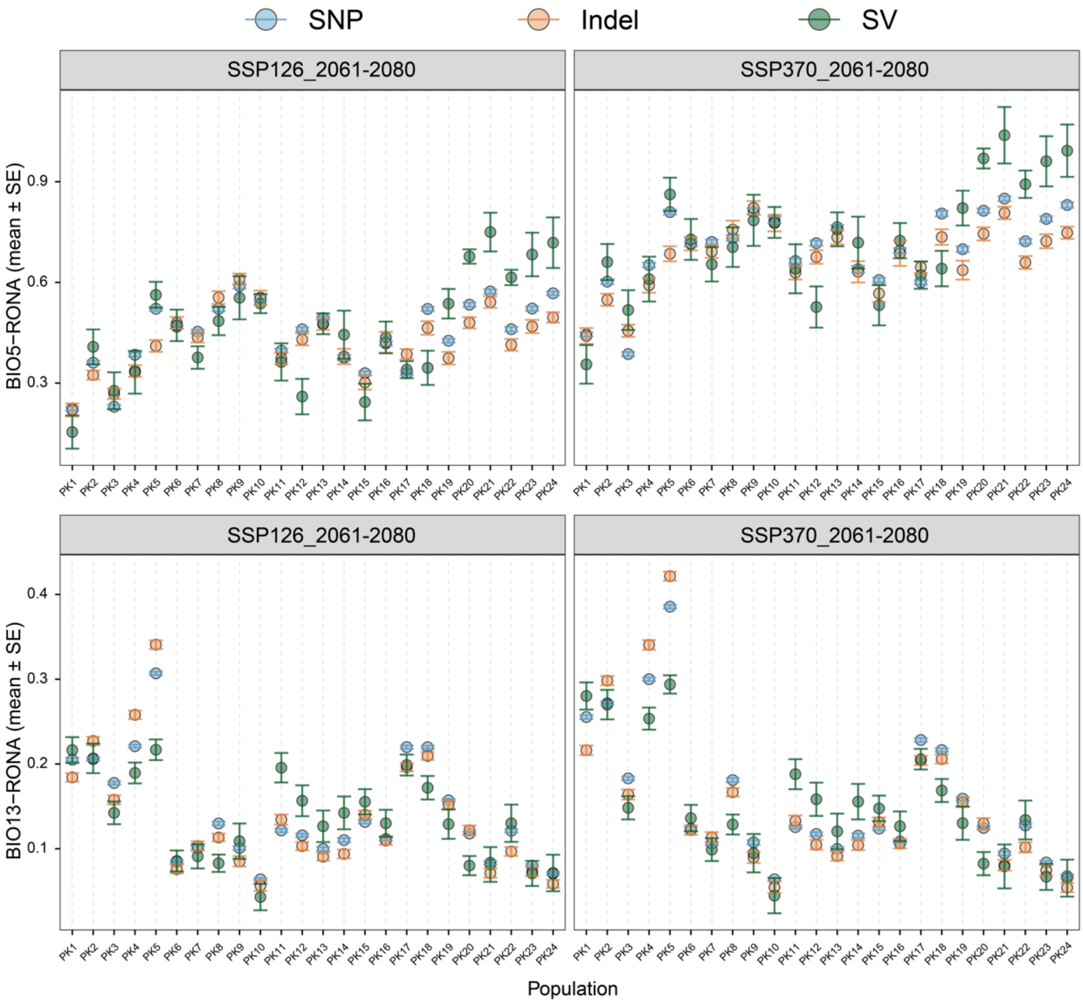

Supplementary Fig. 22. Comparison of RONA estimates for the two representative environmental variables (BIO5 and BIO13) across 24 *P. koreana* populations using the three separated core adaptive datasets of SNPs (BIO5: 245 climatic-associate SNPs; BIO13: 722 climatic-associate SNPs), indels (BIO5: 23 climatic-associate indels; BIO13: 110 climatic-associate indels) and SVs (BIO5: 3 climatic-associate SVs; BIO13: 9 climatic-associate SVs) under two different climate scenarios (SSP126 and SS370) in 2061-2080. Error bar represents standard error of the average RONA (SE). Different colors represent different types of variants (SNPs, indels and SVs).

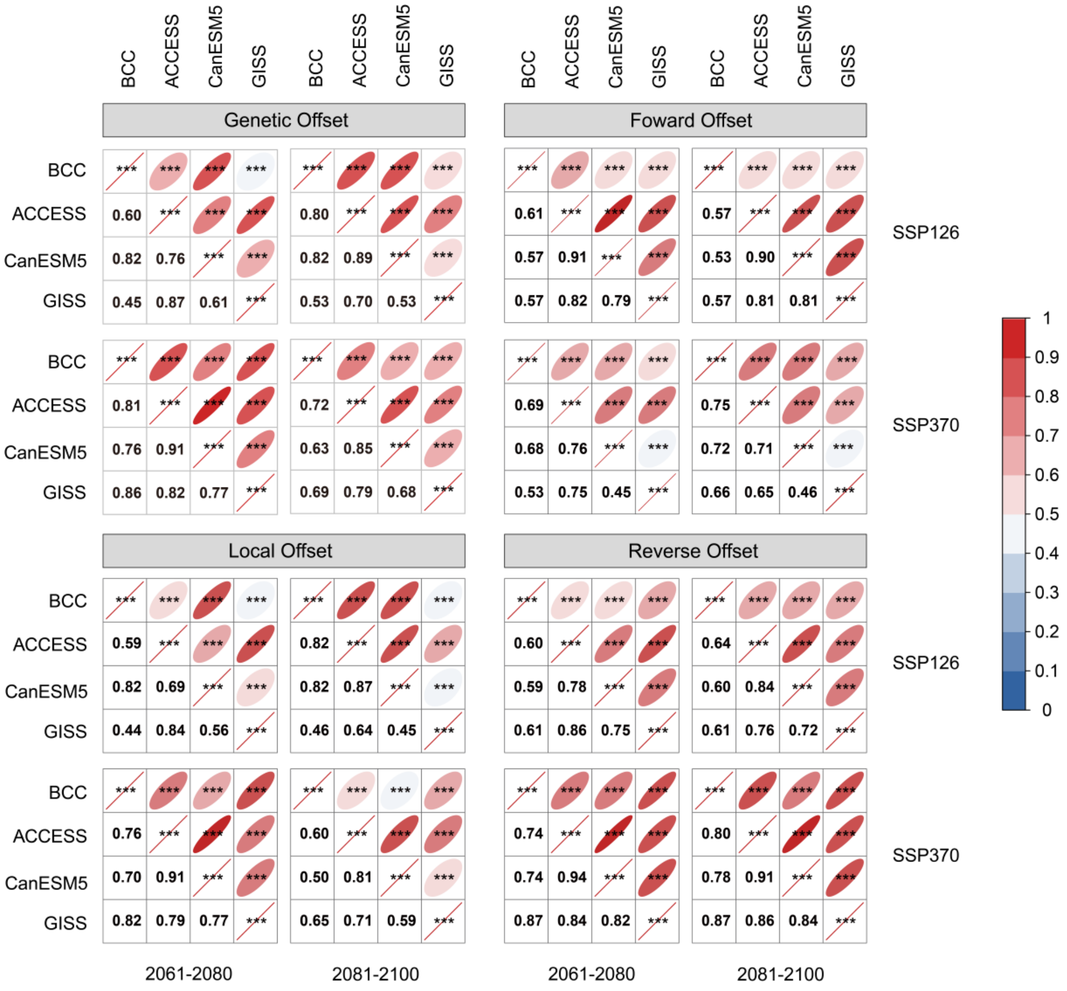

Supplementary Fig. 23. The pairwise correlations of traditional gradient forest-based genetic offsets, and forward, local and reverse offsets when integrating migration into account among four future climate models under two different climate scenarios (SSP 126 and SS370) in 2061-2080 and 2081-2100, respectively. The significance was determined using two-tailed Spearman's correlation analysis. Asterisks indicate significance levels (**P*< 0.05, ***P*< 0.01, ****P*< 0.001). BCC: BCC-CSM2-MR; ACCESS: ACCESS-CM2; GISS: GISS-E2-1-G.

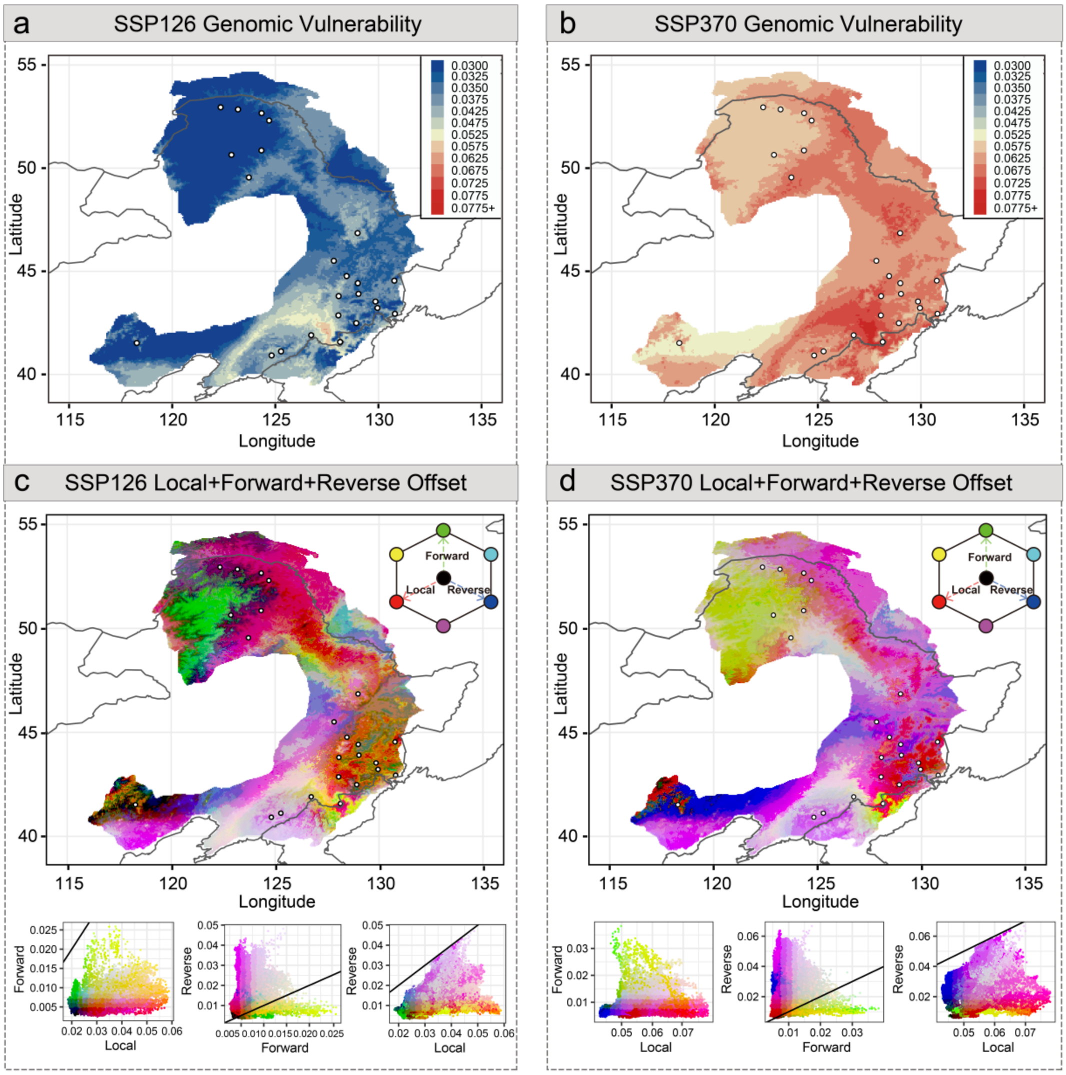

Supplementary Fig. 24. Predicted genetic offsets to future climate change under SSP126 and SSP370 in 2081-2100. a,b, Map of the gradient forest predicted genetic offset averaged across four climate models across the distribution of *P. koreana* (n=60,000 grids) under two scenarios of shared socioeconomic pathways SSP126 (**a**) and SSP370 scenarios (**b**) in 2081-2100. The color scale from blue to red refers to increasing values of genetic offset, and points on map reflect the 24 sampled populations. **c,d,** RGB map of local (red), forward (green) and reverse (blue) offsets throughout the range of *P. koreana* (n=60,000 grids) under SSP126 (**c**) and SSP370 (**d**) scenarios in 2081-2100. Brighter cells (closer to white) have relatively high values along each of the three axes, while darker cells (closer to black) have relatively lower values. Lower panels are the bivariate scattergrams of (**c**) and (**d**) with 1:1 lines.

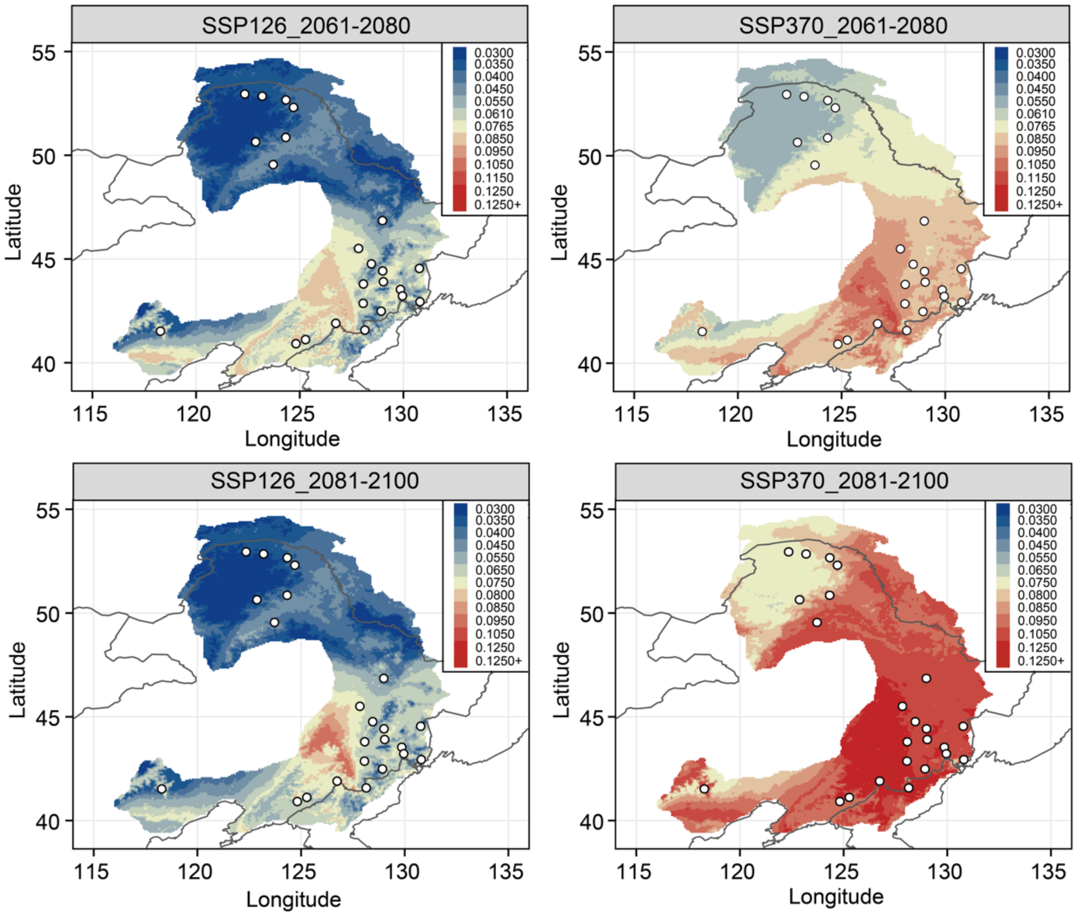

Supplementary Fig. 25. Maps of genomic offset (averages across the four future climate models) calculated by gradient forest-based approach using 6 uncorrelated environmental variables (BIO1, BIO3, BIO5, BIO13, BIO15, BIO19) across the natural distribution of *P. koreana*. The color scale from blue to red refers to the increasing of genetic offset.

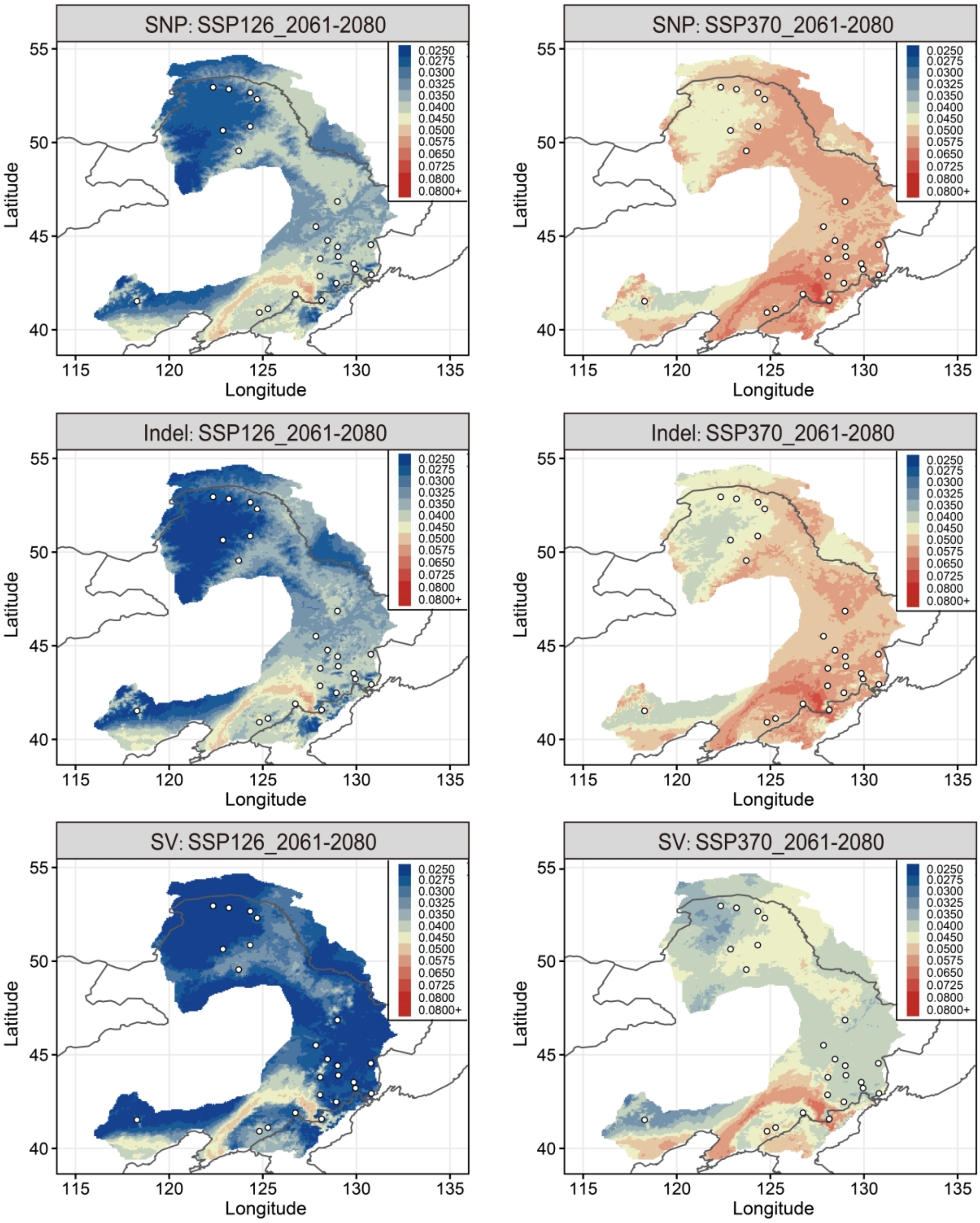

Supplementary Fig. 26. Maps of genomic offset (averages across the four future climate models) calculated by gradient forest-based approach across the natural distribution of *P. koreana* using independent datasets of three types of adaptive variants (SNPs, indels and SVs) detected by both LFMM and RDA. The color scale from blue to red refers to the increasing of genetic offset.

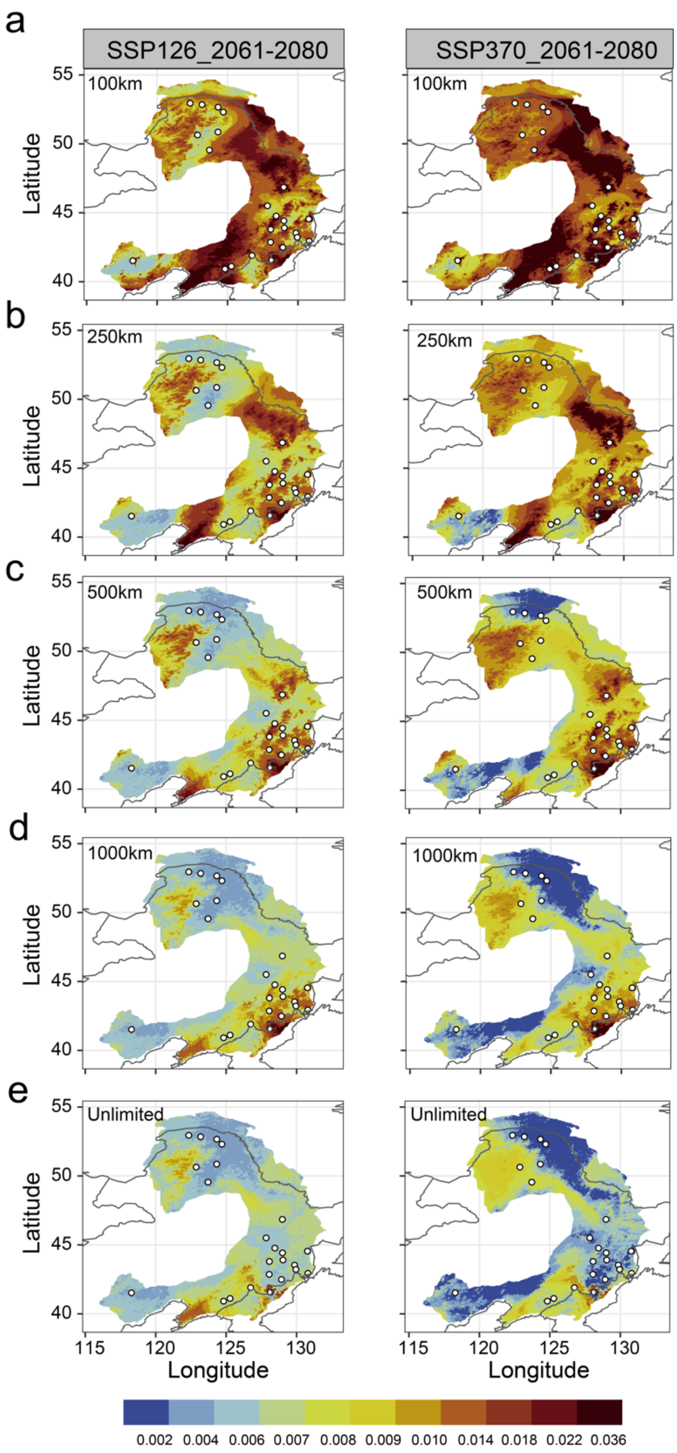

Supplementary Fig. 27. Effect of search distance on forward offset from gradient forest for SSP126 (first column) and SSP370 (second column) in 2061-2080. Distance classes included (**a**) 100 km, (**b**) 250 km, (**c**) 500 km, (**d**) 1000 km, and (**e**) unlimited.

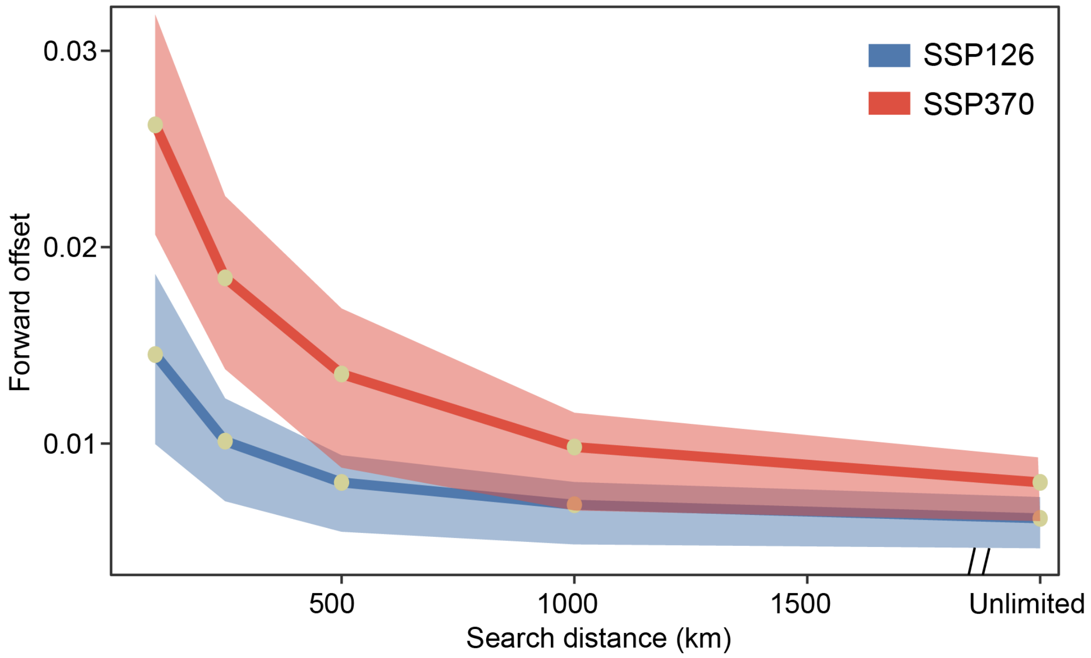

Supplementary Fig. 28. Effect of search distance on forward offset from gradient forest for SSP126 (blue) and SSP370 (red) in 2061-2080. The bands extend between the 25th and 75th percentiles, and the points are the median values.

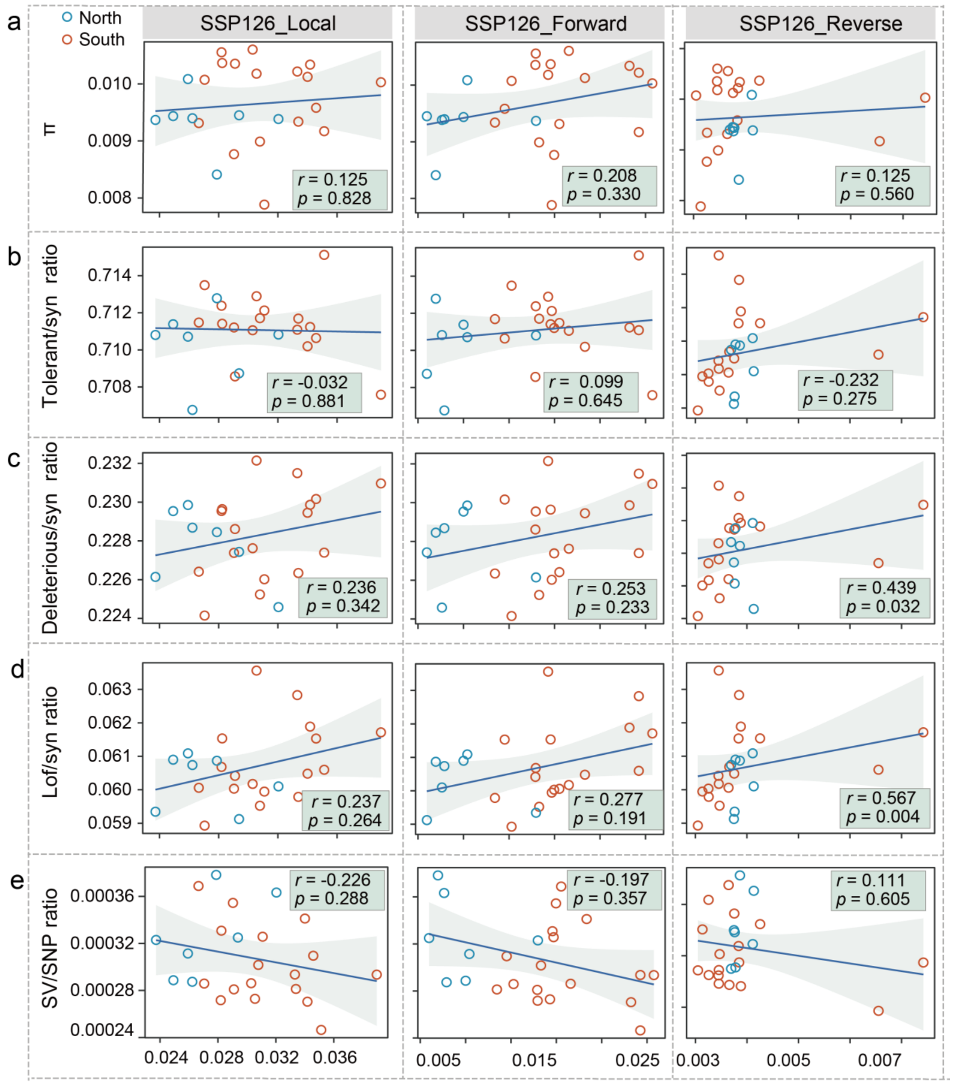

Supplementary Fig. 29. The relationship between multiple different proxies of genetic load (y axis) and genetic offset metrics (x axis) under the SSP126 climate scenarios in 2061-2080. The three columns correspond to the relationship between local offset (left), forward offset (middle) and reverse offset (right) with nucleotide diversity (**a**), the ratio of the number of derived tolerant variants to the number of derived synonymous variants (**b**), the ratio of the number of derived deleterious variants to the number of derived synonymous variants (**c**) and the ratio of the number of loss of function variants to the number of derived synonymous variants (**d**) and the ratio of the number of polymorphic SV variants to the number of polymorphic SNP variants (**e**). Circles colored by blue and red represent the northern and southern populations. The solid blue line denotes the best-fit linear regression line between offsets and genetic loads. The light-blue shades represent the 95% confidence interval.

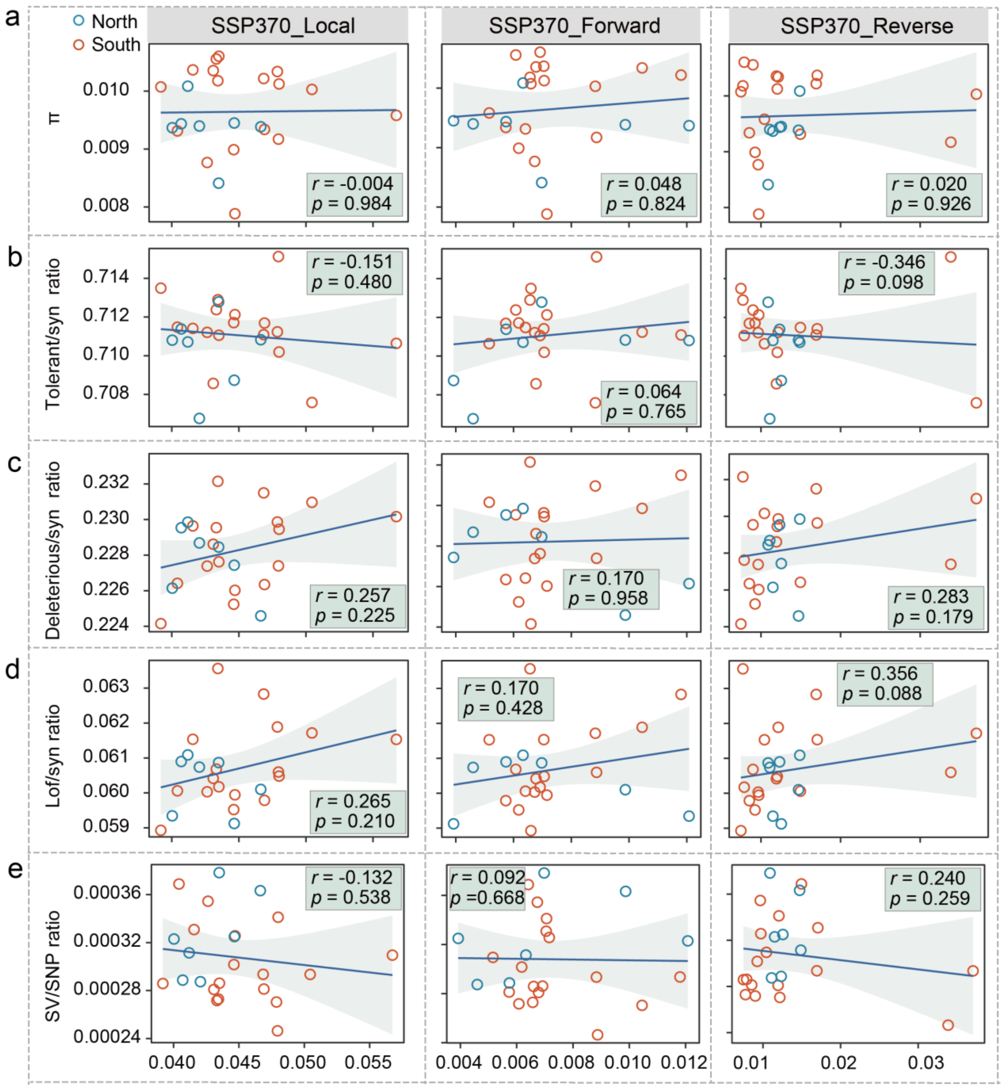

Supplementary Fig. 30. The relationship between multiple different proxies of genetic load (y axis) and genetic offset metrics (x axis) under the SSP370 climate scenarios in 2061-2080. The three columns correspond to the relationship between local offset (left), forward offset (middle) and reverse offset (right) with nucleotide diversity (**a**), the ratio of the number of derived tolerant variants to the number of derived synonymous variants (**b**), the ratio of the number of derived deleterious variants to the number of derived synonymous variants (**c**) and the ratio of the number of loss of function variants to the number of derived synonymous variants (**d**) and the ratio of the number of polymorphic SV variants to the number of polymorphic SNP variants (**e**). Circles colored by blue and red represent the northern and southern populations. The solid blue line denotes the best-fit linear regression line between offsets and genetic loads. The light-blue shades represent the 95% confidence interval.

Supplementary Table 1. Statistics of sequencing reads.

Platform	Total reads	Total bases	Coverage (x)
Nanopore	1,938,650	42,419,739,676	105.68
Illumina	198,806,712	2,982,1006,800	74.29
Hi-C	366,626,810	54,994,021,500	137.00

Supplementary Table 2. Illumina sequencing data statistics.

**sequencing data statistics**	
Total reads	198,806,712
Total bases	29,821,006,800
Clean reads	196,862,480
Clean bases	27,377,255,469
Q20 rate(%)	96.65
Q30 rate(%)	90.79
GC(%)	35.12

Supplementary Table 3. Nanopore sequencing data statistics.

**sequencing data statistics**	
Reads data (bp)	42,419,739,676
Reads number	1,938,650
Reads mean length (bp)	21,881
Reads max length (bp)	214,618
Reads N50 (bp)	28,921
>10kb (%)	80.08
>20kb (%)	47.70
>40kb (%)	11.06

Supplementary Table 4. Mapping summary of Hi-C data.

**Hi-C data**	
Raw Paired-end Reads	366,626,810
Clean Paired-end Reads	361,786,732 (98.68%)
Clean Base(bp)	54,223,719,643
Clean Q30 Base Rate (%)	92.00
Clean Pair-end Reads	180,893,366
Unmapped Paired-end Reads	4,371,524 (2.42%)
Paired-end Reads with Singleton	32,010,746 (17.70%)
Multi Mapped Paired-end Reads	30,347,997 (16.78%)
Unique Mapped Paired-end Reads	114,163,099 (63.11%)
Valid reads of unique mapping reads (%)	86.05
Valid reads of clean reads (%)	54.31

Supplementary Table 5. Scaffolding of contigs based on Hi-C data.

Pseudo-chromosome	Size (Contig number)
**LG01**	51,125,891 (15)
**LG02**	26,717,773 (7)
**LG03**	25,475,328 (5)
**LG04**	25,429,882 (7)
**LG05**	24,496,347 (10)
**LG06**	22,536,972 (5)
**LG07**	22,306,471 (9)
**LG08**	19,875,049 (6)
**LG09**	19,170,182 (8)
**LG10**	19,169,738 (6)
**LG11**	17,803,069 (11)
**LG12**	17,266,452 (8)
**LG13**	16,661,543 (5)
**LG14**	16,606,995 (5)
**LG15**	15,942,094 (7)
**LG16**	15,894,631 (8)
**LG17**	15,420,218 (3)
**LG18**	14,612,902 (5)
**LG19**	13,424,669 (3)
**Total**	399,936,206 (133)

Supplementary Table 6. Evaluation of assembly (Nonredundant and Noncontaminated Genome) completeness with respect to gene space using BUSCO.

**BUSCO**	**Number (Percentage)**
Complete BUSCOs (C)	1,579 (97.83%)
Complete and single-copy BUSCOs(S)	1,310 (81.16%)
Complete and duplicated BUSCOs(D)	269 (16.67%)
Fragmented BUSCOs(F)	13 (0.81%)
Missing BUSCOs(M)	22 (1.36%)
Total BUSCO groups searched	1,614 (100%)

Supplementary Table 7. Transposon elements (TE) annotation of *P. koreana* genome.

**Classification**	**Count**	**Length(bp)**	**Percentage of genome (%)**
**LTR**			
Copia	17,024	12,757,941	3.18
Gypsy	47,866	35,192,597	8.77
unknown	39,979	15,189,643	3.78
**nonLTR**			
DIRS_YR			
LINE_element	1,091	772,846	0.19
unknown	191	109,452	0.03
**TIR**			
CACTA	17,212	6,832,438	1.70
Mutator	21,654	7,879,959	1.96
PIF_Harbinger	8,225	2,464,697	0.61
Tc1_Mariner	1,849	852,149	0.21
hAT	12,179	4,521,301	1.13
polinton			
**nonTIR**			
helitron	121,956	49,275,078	12.28
**repeat_region**	47,380	13,429,450	3.35
**Total**	336,606	149,277,551	37.19

Supplementary Table 8. Statistics of the annotation of protein-coding genes of *P. koreana* genome.

**Gene annotation statistics**	
Gene number	37,072
Average gene length (bp)	3779.29
Mean exon number per mRNA	5.05
Mean CDS number per mRNA	4.92
Average CDS length (bp)	1136.04
Average intron length (bp)	1997.64
Average single exon length	318.16
Average single CDS length	230.77
Average single intron length	492.81

Supplementary Table 9. Functional annotation of protein-coding genes of *P. koreana* genome.

**Dataset**	**Number(percentage)**
Pfam	26,037(70.23%)
Interproscan	33,307(89.84%)
KEGG	11,253(30.35%)
NR	34,300(92.52%)
Swiss-Prot	26,971(72.75%)
KOG	30,774(83.01%)
COG	12,532(33.80%)
Tremble	34,802(93.88%)
GO	27,460(74.07%)
Unannotated	1,692(4.56%)
Total	37,072

Supplementary Table 10. Statistics of the annotated non-coding RNA.

**RNA type**		**number**
miRNA		3240
tRNA		669
SnRNA		91
SnoRNA		516
rRNA	5S rRNA	56
	5.8S rRNA	6
	18S rRNA	14
	28S rRNA	14

Supplementary Table 11. Environmental variables used in this study derived from WorldClim.

**Code**	**Variable**
Temperature-related	
BIO1	Annual Mean Temperature
BIO2	Mean Diurnal Range
BIO3	Isothermality (BIO2/BIO7)
BIO4	Temperature Seasonality
BIO5	Maximum Temperature of Warmest Month
BIO6	Minimum Temperature of Coldest Month
BIO7	Temperature Annual Range (BIO5-BIO6)
BIO8	Mean Temperature of Wettest Quarter
BIO9	Mean Temperature of Driest Quarter
BIO10	Mean Temperature of Warmest Quarter
BIO11	Mean Temperature of Coldest Quarter
Precipitation-related	
BIO12	Annual Precipitation
BIO13	Precipitation of Wettest Month
BIO14	Precipitation of Driest Month
BIO15	Precipitation Seasonality
BIO16	Precipitation of Wettest Quarter
BIO17	Precipitation of Driest Quarter
BIO18	Precipitation of Warmest Quarter
BIO19	Precipitation of Coldest Quarter

Supplementary Table 12. The number and proportion of the functional effects of the environmental-associated variants identified by LFMM, both LFMM and RDA relative to the whole genome level.

	**LFMM**	**LFMM&RDA**	**Genome**
3'UTR	60(1.75%)	28(1.57%)	181,752(3.05%)
5'UTR	81(2.36%)	55(3.09%)	83,429(1.40%)
Upstream	724(21.08%)	284(15.96%)	1,144,408(19.24%)
Downstream	509(14.82%)	278(15.63%)	792,457(13.32%)
Nonsynonymous	97(2.82%)	56(3.15%)	148,847(2.50%)
Synonymous	64(1.86%)	35(1.97%)	126,672(2.13%)
Intron	382(11.12%)	187(10.51%)	682,145(11.47%)
Intergenic	1,501(43.70%)	850(47.78%)	2,767,838(46.52%)
Total	3,438(99.51%)	1,773(99.66%)	5,927,548(99.63%)

Supplementary Table 13. Summary of some functional important genes with relatively high numbers of environmental-associated variants detected.

PK_GENE	Ptri_gene	Atha_gene	symbol	Chromosome	Associated variants	Related environment variables
Pokor00456	Potri.001G412077	AT4G27280	CMI1	LG01	14	BIO15
Pokor01235	Potri.001G330900	AT3G01910	SOX	LG01	9	BIO13,16,18
Pokor01408	Potri.001G312300	AT1G17840	ABCG11	LG01	4	BIO5
Pokor02467	Potri.001G205300	AT5G09420	TOC64-V	LG01	5	BIO3
Pokor09179	Potri.005G052800	AT4G14640	CAM8	LG03	3	BIO2
Pokor10559	Potri.002G172800	AT4G23980	ARF9	LG04	7	BIO13,16,18
Pokor10689	Potri.002G159300	AT2G45880	BMY4	LG04	17	BIO12,13,14,16,17,18,19
Pokor11081	Potri.014G016300	AT5G67500	VDAC2	LG04	2	BIO12,13,14,16,17,18,19
Pokor12242	Potri.002G005000	AT2G33620	AHL10	LG04	3	BIO13,18
Pokor12247	Potri.002G004500	AT2G33590	CRL1	LG04	31	BIO12,13,14,16,17,18,19
Pokor12248	Potri.002G004100	AT2G33590	CRL1	LG04	73	BIO12,13,14,16,17,18,19
Pokor13059	Potri.004G085500	AT5G37630	EMB2656	LG05	13	BIO12,14,17,18,19
Pokor13254	Potri.004G103200	AT5G39050	PMAT1	LG05	13	BIO13
Pokor14452	Potri.004G230100	AT3G18990	VRN1	LG05	38	BIO14,17,19
Pokor17228	Potri.003G039475	AT3G13860	HSP60-3A	LG07	62	BIO5,8,12,13,14,16,17,18,19
Pokor18546	Potri.003G168700	AT5G14090	LAZY1	LG07	8	BIO13,16,18
Pokor18547	Potri.003G168800	AT1G27980	DPL1	LG07	12	BIO13,16,18
Pokor19962	Potri.014G111400	AT2G46970	PIL1	LG08	3	BIO15
Pokor21575	Potri.011G151700	AT3G15850	FAD5	LG09	82	BIO5,8,10,12,13,14,16,17,18,19
Pokor21577	Potri.011G152200	AT1G32900	GBSS1	LG09	20	BIO8,10,12,13,14,16,17,18,19
Pokor22025	Potri.011G041700	AT4G05070	WIP2	LG09	4	BIO5
Pokor23027	Potri.008G079700	AT5G16300	COG1	LG10	6	BIO13
Pokor25841	Potri.007G138800	AT3G60030	SPL12	LG12	87	BIO12,13,14,16,17,18,19
Pokor27800	Potri.013G067500	AT1G08810	MYB60	LG13	3	BIO13
Pokor28275	Potri.013G120800	AT4G35160	ASMT1	LG13	6	BIO8,10,12,13,16,17,18,19
Pokor29050	Potri.018G051300	AT5G07990	TT7	LG14	5	BIO5
Pokor29543	Potri.018G101300	AT2G23950	CIK2	LG14	20	BIO1,2,4,6,7,9,11
Pokor32219	Potri.012G097300	AT1G20960	EMB1507	LG16	27	BIO5

Supplementary Table 14. Sequence of primers used for qRT-PCR test on abiotic stress

Symbol	Forward Primer	Reverse Primer
HSP60-3A(Pokor17228)	CTAAGCCCAGATAGATACTTGC	ATTTCATAGGAATGGACCGAC
CRL1 (Pokor12447)	CGAGTTGTTTTGAAACCGAA	TAGGTGCTTATGAAAATACCAG
UBQ-10	CCAAGCCCAAGAAGATCAAGC	GCACCGCACTCAGCATTAGG
