# Supplementary material for: Genomic insights into local adaptation and future climate-induced vulnerability of a keystone forest tree in East Asia
Source: Nat Commun. 2022 Nov 1;13:6541. doi: 10.1038/s41467-022-34206-8 (PMC9626627; doi:10.1038/s41467-022-34206-8)
Supplement: Supplementary file 3 — Description of Additional Supplementary Files [file 41467_2022_34206_MOESM3_ESM.pdf]

Description of Additional Supplementary Files

File Name: Supplementary Data 1
Description: Geographical sampling information and summary statistics of whole-genome resequencing data for samples used in this study.

File Name: Supplementary Data 2
Description: Detailed information of environmental associated variants identified in this study.

File Name: Supplementary Data 3
Description: Risk of nonadaptedness (RONA) calculated using the four climate models for environmental variables of BIO1-BIO19 across the 24 natural populations of P. koreana under climate scenarios of SSP126 and SSP370 in 2061-2080 and 2081-2100.
