# Supplementary material for: Genomic insights into local adaptation and future climate-induced vulnerability of a keystone forest tree in East Asia
Source: Nat Commun. 2022 Nov 1;13:6541. doi: 10.1038/s41467-022-34206-8 (PMC9626627; doi:10.1038/s41467-022-34206-8)
Supplement: Supplementary file 7 — Reporting Summary [file 41467_2022_34206_MOESM7_ESM.pdf]

nature portfolio

Corresponding author(s): DBPR NCOMMS-22-10366

Last updated by author(s): Oct 7, 2022

Reporting Summary

Nature Portfolio wishes to improve the reproducibility of the work that we publish. This form provides structure for consistency and transparency in reporting. For further information on Nature Portfolio policies, see our Editorial Policies and the Editorial Policy Checklist.

Statistics

For all statistical analyses, confirm that the following items are present in the figure legend, table legend, main text, or Methods section.

n/a	Confirmed	
☐	☒	The exact sample size (*n*) for each experimental group/condition, given as a discrete number and unit of measurement
☐	☒	A statement on whether measurements were taken from distinct samples or whether the same sample was measured repeatedly
☐	☒	The statistical test(s) used AND whether they are one- or two-sided<br>*Only common tests should be described solely by name; describe more complex techniques in the Methods section.*
☐	☒	A description of all covariates tested
☐	☒	A description of any assumptions or corrections, such as tests of normality and adjustment for multiple comparisons
☐	☒	A full description of the statistical parameters including central tendency (e.g. means) or other basic estimates (e.g. regression coefficient) AND variation (e.g. standard deviation) or associated estimates of uncertainty (e.g. confidence intervals)
☐	☒	For null hypothesis testing, the test statistic (e.g. *F*, *t*, *r*) with confidence intervals, effect sizes, degrees of freedom and *P* value noted<br>*Give P values as exact values whenever suitable.*
☒	☐	For Bayesian analysis, information on the choice of priors and Markov chain Monte Carlo settings
☐	☒	For hierarchical and complex designs, identification of the appropriate level for tests and full reporting of outcomes
☐	☒	Estimates of effect sizes (e.g. Cohen's *d*, Pearson's *r*), indicating how they were calculated

Our web collection on statistics for biologists contains articles on many of the points above.

Software and code

Policy information about availability of computer code

Data collection	Environmental data were extracted from GPS coordinates of sampling sites from publicly available sources cited in the manuscript. No software was used to collect the genotype data.
Data analysis	NextDenovo v2.0-beta.1;Nextpolish v1.0.5;purge_haplotigs v1.1.1;Bowtie2 v2.3.2;HiC-Pro v2.11.4;bwa v0.7.12;EDTA v1.9.3;RepeatMasker v4.1.0;HISAT v2.2.1;StringTie;PASA v2.4.1;Augustus v3.3.2;EvidenceModelerv1.1.1; tRNAscan-SE v2.0.7;Trimmomatic v0.36;GATK v.4.0.5.1;DELLY v0.8.3;Maxent v.3.3.3;PLINK v1.90;vegan 2.6-2;VCFtools v0.1.15;FigTree v.1.4.4;PopLDdecay v.3.40;PSMC;LEA v3.3.2;selscan v.1.3.0;Picard v.2.18.11

For manuscripts utilizing custom algorithms or software that are central to the research but not yet described in published literature, software must be made available to editors and reviewers. We strongly encourage code deposition in a community repository (e.g. GitHub). See the Nature Portfolio guidelines for submitting code & software for further information.

Data

Policy information about availability of data

All manuscripts must include a data availability statement. This statement should provide the following information, where applicable:

- Accession codes, unique identifiers, or web links for publicly available datasets
- A description of any restrictions on data availability
- For clinical datasets or third party data, please ensure that the statement adheres to our policy

All data needed to evaluate the conclusions in the paper are present in the paper and/or the Supplementary Materials. All sequencing data, including the genome assembly (Nanopore long reads, Illumina reads of whole-genome sequencing, transcriptomes, and Hi-C reads) and whole-genome resequencing data for 230 individuals in this study have been deposited in the National Genomics Data Center (https://ngdc.cncb.ac.cn) under accession number PRJCA008692 [https://

ngdc.cncb.ac.cn/bioproject/browse/PRJCA008692]. The assembled genome and gene annotation files are available at https://zenodo.org/record/7156132. Source data are provided with this paper. All scripts used in this study are available at https://github.com/jingwanglab/Populus_genomic_prediction_climate_vulnerability.

Field-specific reporting

Please select the one below that is the best fit for your research. If you are not sure, read the appropriate sections before making your selection.

☐ Life sciences ☐ Behavioural & social sciences ☒ Ecological, evolutionary & environmental sciences

For a reference copy of the document with all sections, see nature.com/documents/nr-reporting-summary-flat.pdf

Ecological, evolutionary & environmental sciences study design

All studies must disclose on these points even when the disclosure is negative.

Study description	Our study uses genotype data from Populus koreana that was generated from 230 individuals collected from 24 nature populations from East Asia along with environmental data from WorldClim, to reveal the genomic basis of local adaptation to diverse climate variable and predict spatiotemporal responses of P. koreana to future climate change.
Research sample	Populus koreana is one dominant tree species in temperate deciduous forests in East Asia. In this study, we collected 230 individuals from 24 natural populations across its distribution range.
Sampling strategy	A total of 230 individuals were collected from 24 natural populations, representing most natural habitats of P. koreana. Within each population, individuals were sampled after ensuring that sampled individuals were at least 100m apart from each other. The number of individuals sampled from each population ranged from 8-10.
Data collection	Field data were collected by Hongying Zhang and Yupeng Sang. Whole genome resequencing and genome sequencing procedure were described in manuscript.
Timing and spatial scale	Samples were collected in 2019 and used for all analyses. total of 230 individuals were collected from 24 natural populations, representing most natural habitats of P. koreana.
Data exclusions	No data was excluded from analyses.
Reproducibility	Citations of all data sources, and public deposition of whole genome resequencing data and genome sequencing data.
Randomization	The work was not experimental in nature, and did not necessitate random assignment of individuals to treatment groups.
Blinding	This does not apply to our study.

Did the study involve field work? ☐ Yes ☒ No

Reporting for specific materials, systems and methods

We require information from authors about some types of materials, experimental systems and methods used in many studies. Here, indicate whether each material, system or method listed is relevant to your study. If you are not sure if a list item applies to your research, read the appropriate section before selecting a response.

Materials & experimental systems

n/a	Involved in the study	
☒	☐	Antibodies
☒	☐	Eukaryotic cell lines
☒	☐	Palaeontology and archaeology
☒	☐	Animals and other organisms
☒	☐	Human research participants
☒	☐	Clinical data
☒	☐	Dual use research of concern

Methods

n/a	Involved in the study	
☒	☐	ChIP-seq
☒	☐	Flow cytometry
☒	☐	MRI-based neuroimaging
